# Towards mixed physical node reservoir computing: light-emitting synaptic reservoir system with dual photoelectric output

**DOI:** 10.1038/s41377-024-01516-z

**Published:** 2024-08-01

**Authors:** Minrui Lian, Changsong Gao, Zhenyuan Lin, Liuting Shan, Cong Chen, Yi Zou, Enping Cheng, Changfei Liu, Tailiang Guo, Wei Chen, Huipeng Chen

**Affiliations:** 1 Institute of Optoelectronic Display, National & Local United Engineering Lab of Flat Panel Display Technology, Fuzhou University, Fuzhou, 350002 China; 2 Fujian Science & Technology Innovation Laboratory for Optoelectronic Information of China, Fuzhou, 350100 China; 3 Joint School of National University of Singapore and Tianjin University, International Campus of Tianjin University, Fuzhou, 350207 China; 4 Department of Chemistry, National University of Singapore, 3 Science Drive 3, Singapore, 117543 Singapore; 5 Department of Physics, National University of Singapore, 3 Science Drive 3, Singapore, 117543 Singapore

**Keywords:** Optoelectronic devices and components, Photonic devices

## Abstract

Memristor-based physical reservoir computing holds significant potential for efficiently processing complex spatiotemporal data, which is crucial for advancing artificial intelligence. However, owing to the single physical node mapping characteristic of traditional memristor reservoir computing, it inevitably induces high repeatability of eigenvalues to a certain extent and significantly limits the efficiency and performance of memristor-based reservoir computing for complex tasks. Hence, this work firstly reports an artificial light-emitting synaptic (LES) device with dual photoelectric output for reservoir computing, and a reservoir system with mixed physical nodes is proposed. The system effectively transforms the input signal into two eigenvalue outputs using a mixed physical node reservoir comprising distinct physical quantities, namely optical output with nonlinear optical effects and electrical output with memory characteristics. Unlike previously reported memristor-based reservoir systems, which pursue rich reservoir states in one physical dimension, our mixed physical node reservoir system can obtain reservoir states in two physical dimensions with one input without increasing the number and types of devices. The recognition rate of the artificial light-emitting synaptic reservoir system can achieve 97.22% in MNIST recognition. Furthermore, the recognition task of multichannel images can be realized through the nonlinear mapping of the photoelectric dual reservoir, resulting in a recognition accuracy of 99.25%. The mixed physical node reservoir computing proposed in this work is promising for implementing the development of photoelectric mixed neural networks and material-algorithm collaborative design.

## Abstract (web summary)

This manuscript proposes a photoelectric dual-output mixed physical node reservoir system. It achieves higher handwriting digit recognition accuracy and use the photoelectric output characteristics to achieve multichannel image recognition.

## Graphical abstract

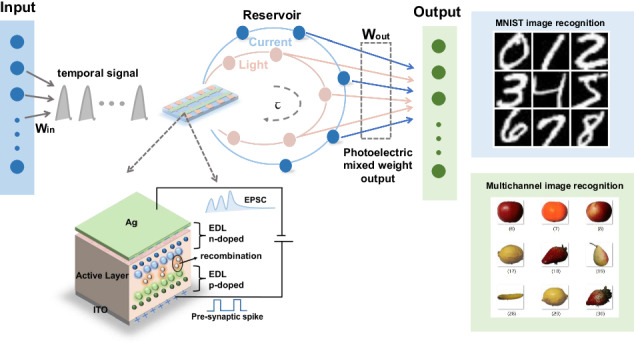

## Introduction

With the development of artificial intelligence technology, various artificial hardware neural networks such as recursive neural networks^1^, convolutional neural networks^2^, and spiking neural networks^3^ have been proposed to meet the efficient processing and recognition of massive data. Recently, the physical reservoir computing (PRC) network has received extensive attention due to its advantages of no need to establish the isomorphic relationship between hardware and algorithm, low training cost, and has become one of the main neuromorphic computing paradigms for high-dimensional and nonlinear computing^4–7^. Many physical reservoirs have been demonstrated to enable reservoir computing (RC), such as memristors^8^, atomic switching networks^9^, silicon photonics^10^, and spintronic oscillators^11^. In particular, memristor-based RC systems have been widely reported in RC in recent years due to simple structure, high degree of freedom system response, and high integration^12–14^.

**Fig. 1:**
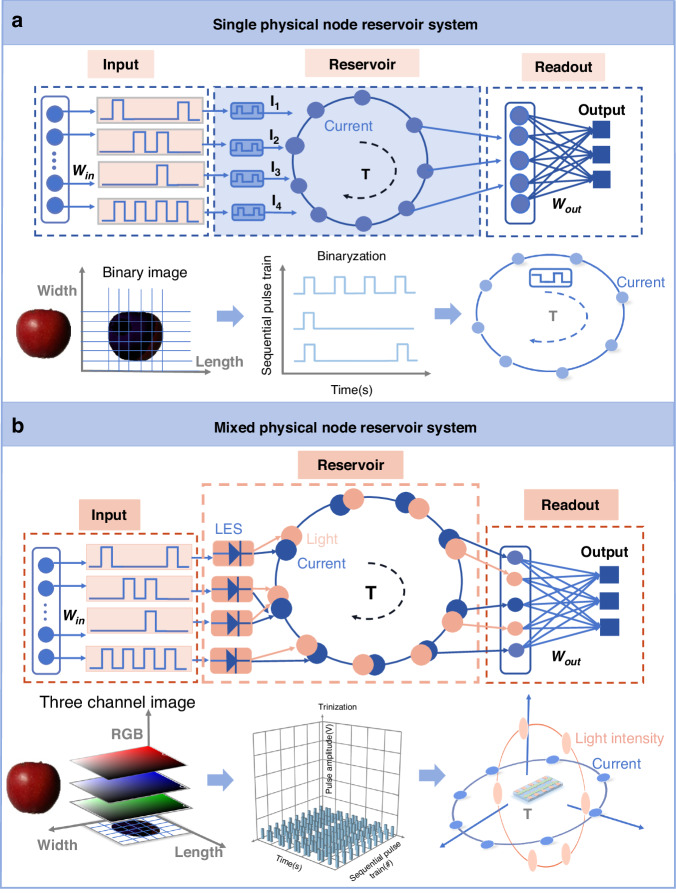
Single physical node reservoir computing system schematic and mixed physical node reservoir computing system schematic. **a** Schematic illustration of a memristor-based RC system, which builds physical node by reading current. The RC system maps the time sequence pulse signal to the current to realize the recognition of the binary image. **b** Schematic diagram of a mixed physical node RC system based on LES, which builds mixed physical node by two signals of current and light intensity. The RC system can realize the recognition of multichannel image by mapping time sequence pulse signal to current and mapping pulse amplitude signal to light intensity

However, current work on dynamic memristor-based RC systems often focuses on using the output current or conductance state of the devices as the dynamically evolving reservoir state to map input temporal signals^15–18^. This approach overlooks some important issues that are critical for RC. On one hand, using electrical signals as the single node in the reservoir can lead to issues of overlapping and interference in the reservoir state during the mapping of complex sequential signals. This often results in difficulties in effectively extracting the spatiotemporal characteristics of complex information, thus limiting the richness of the reservoir state in physical reservoir computing. Although some work has proposed extracting features of input signals by using mixed modalities such as light and electricity, the reservoir in those cases still relies on a single electrical evolution as the reservoir node, which cannot be further optimized^12,15,17,19^. On the other hand, using a single-node strategy for the reservoir makes it challenging to meet the requirements of mapping multiple features of high-dimensional data in the real world, which puts forward higher requirements for the parallel processing of data. For instance, when working with multichannel pictures, in addition to two dimensions of height and width, there are three RGB color channels^20,21^ (Fig. 1b). However, the previously reported memristor reservoir system only consists of a single type of physical node (Fig. 1a), and they only map the input signal through a single feature, which makes the recognition of the memristor reservoir system only from the shape of the image content, and cannot realize the parallel processing of multi-feature information^8,19,20^. Therefore, it is crucial to develop new dynamic memristors with mixed nodes based on novel device physics and material designs in order to simultaneously meet the requirements of a rich reservoir state and parallel processing of high-dimensional data.

In this work, we report a mixed physical node reservoir computing system based on artificial light-emitting synapses (LES) (Fig. 1b). The device not only has the performance of electrical synapses, which can map the input timing signal with the short-term memory current induced by electrical pulses but also exhibits the behavior of luminescent synapses to map the amplitude of input signals in the form of dynamic emission. Therefore, the device as a physical reservoir can construct a 2D reservoir state space to extract the spatiotemporal characteristics of the temporal signal more effectively and achieve more than 97.22% recognition accuracy in the image classification task based on the MNIST dataset. More importantly, with the help of short-term memory current and dynamic luminescence characteristics with nonlinear optical effects, the signal mapping method of different physics makes the device can also be used in the reservoir computing of multi-feature fusion recognition to solve the problem of low reservoir state richness caused by the single reservoir node of traditional dynamic memristors, which improves the recognition accuracy of multichannel image recognition from 93.16% to 99.25%. This work proposes a new idea for developing neural hardware for complex reservoir computing networks and has great potential in the development of a new generation of artificial neuromorphic hardware.

## Result

### Characterization of artificial synaptic properties in LES

**Fig. 2:**
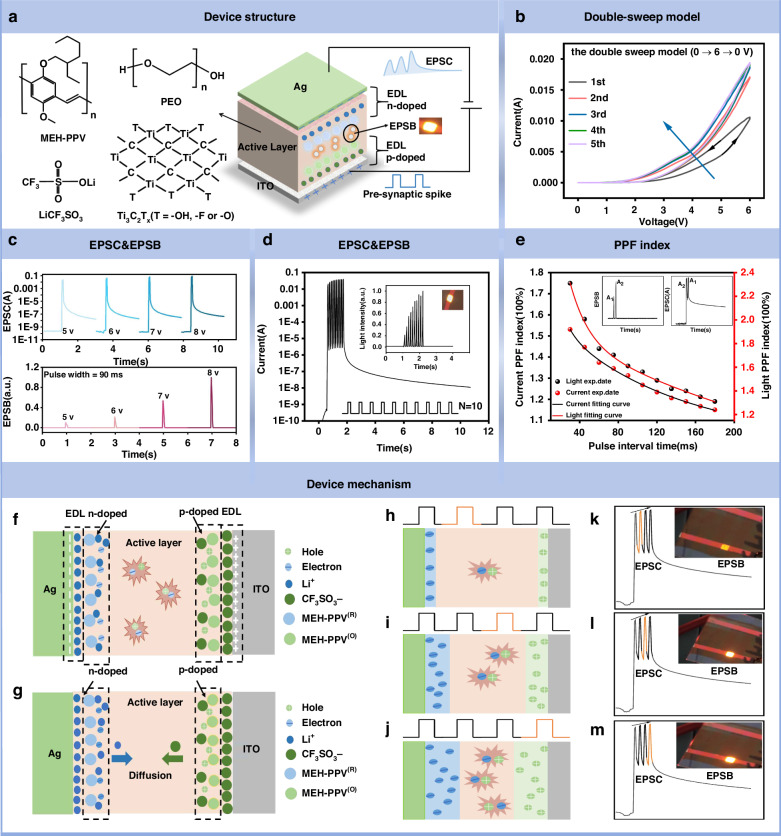
Characterization of artificial synaptic properties in LES and its working mechanism. **a** The chemical structural formula of MEH-PPV, PEO, LiCF_3_SO_3_, and MXene. Schematic diagram of light-emitting artificial synapse. **b** I–V characteristics of the device measured in the consecutive double-sweep model (0→6→0 V). **c** Excitatory postsynaptic current (EPSC) and brightness (EPSB) triggered by presynaptic spikes with different voltages (5–8 V). **d** EPSC and EPSB responses on the stimulation of 10 continuous presynaptic spikes (6 V, 90 ms) with a 60 ms interval. **e** Current and light intensity PPF index (A_2_/A_1_×100%, solid sphere) as a function of the presynaptic spike internal Δt, the continuous line is the result of fitting using a single exponential decay function. **f** Electrochemical reaction process inside the device during the electric pulse. **g** Ion diffusion process inside the device after removing the electric pulse. **h**–**j** Dynamic p-i-n area diffusion process under continuous electrical pulse stimulation. **k**–**m** Optical pictures of the transient brightness enhancement of the device pulse stimulation

Figure 2a shows the 3D structure of the artificial light-emitting synaptic device as a physical reservoir. The device is a three-layer structure device composed of ITO/ active layer /Ag. The active layer is composed of poly[2-methoxy-5-(2-ethylhexyloxy)-1,4-phenylenevinylene] (MEH-PPV), the ion transport matrix poly (ethylene oxide) (PEO), the ion transport matrix poly (ethylene oxide) (LiCF_3_SO_3_) and the dopant MXene (Ti_3_C_2_T_X_), which are used as luminescent polymer semiconductors, ion transport substrates, electrolyte salts and dopants. The chemical structural formula of MEH-PPV, PEO, LiCF_3_SO_3_, and Ti_3_C_2_T_X_ are shown in Fig. 2a. The I–V characteristics of the LES applied for five consecutive dual positive voltage sweeps (0→6→0 V) are displayed in Fig. 2b. The device’s I–V cycle exhibits a counterclockwise hysteresis, which is explained by the active layer’s ion relaxation effect^22^. Additionally, the LES generates red light with a wavelength of 540 nm as its conductance steadily rises and becomes saturated with an increasing number of voltage sweeps (Fig. S1). Figure 2c shows the trend of excitatory postsynaptic current (EPSC) and excitatory postsynaptic brightness (EPSB) with the increase of pulse voltage (5–8 V, 90 ms). When the pulse voltage was raised from 5 V to 8 V, the attenuation time lengthened, indicating memory-enhancing capabilities akin to those of biological synapses. Furthermore, the peak value of EPSB increased by almost ten times when the pulse voltage was raised from 5 V to 8 V, and transient luminance gradually increased. (The illustration in Fig. 2c). The variations in postsynaptic current and luminescence intensity brought about by ten electrical pulses with a 6 V pulse amplitude, a 60 ms pulse duration, and a 60 ms pulse interval are displayed in Fig. 2d. As the number of pulses increases, so does the postsynaptic current. The insertion plot makes it evident that the transitory luminance increases with the number of applied pulses by displaying the corresponding postsynaptic luminous intensity. This finding demonstrates that synaptic enhancing behavior is also shown by the transitory optical signal generated by LES. In addition, by applying pulses of various widths and frequencies, the spike duration time-dependent plasticity (SDDP) (Figs. S2 and S3) and the spike rate-dependent plasticity (SRDP) (Figs. S4 and S5) were achieved. Fig. S6 displays the maximum EPSC value and EPSC gain measured at various frequencies, whereas Fig. S7 displays the highest EPSB value and EPSB gain observed at various frequencies. The spike number-dependent plasticity (SNDP) (Figs. S8 and S9) was examined by progressively increasing the number of pulses. The PPF behavior simulated by the artificial luminous synapse is shown in Fig. S10, where two consecutive electrical pulses (6 V, 30 ms) are applied to the ITO electrode at 30 ms intervals. The EPSC peak stimulated by the second pulse was 1.23 times higher than that stimulated by the first pulse. At the same time, the EPSB peak stimulated by the second pulse was 1.68 times that of the first pulse. The PPF index is calculated by the following formula:

1 $${{PPF}}_{{index}}=\frac{{A}_{2}-{A}_{1}}{{A}_{1}}$$

Figure 2e fits the attenuation process of the PPF index of EPSC and EPSB through the following single exponential function:

2 $${{PPF}}_{{index}}={B}_{0}+{e}^{-\frac{\triangle T}{\tau }}+{B}_{1}$$

As the pulse interval gradually increases, the PPF index decreases to close to 100%.

### The mechanism of the synaptic behavior of LES

After an electrical pulse is applied to the device, the mobile anions move toward the anode and the cations move toward the cathode, forming an electric double layer (EDL) at the interface between the active layer and the electrode (Fig. 2f). When the voltage is higher than the semiconductor band gap of MEH-PPV (V pre > E g/e), MEH-PPV is oxidized to an oxidation state (MEH-PPV^(O)^) or reduced to a reduced state (MEH-PPV^(R)^), the charges are injected through the barrier and then electrostatic compensated by Li^+^ and CF_3_SO_3_^−^^23–25^. The regions where electron-hole recombination occurs (n-doped and p-doped) are formed by the electrochemical redox^26–28^ (Fig. 2g). The role of MXene is to promote the dissociation of LiCF_3_SO_3_ in PEO, thus, the ion transport in the polymer matrix is greatly promoted^29–33^. With the increase of the number of electrical pulses, the n and p doping regions continue to expand (Fig. 2h–j), and the channel current density increases accordingly, resulting in the enhancement of EPSC. At the same time, an increase in electron-hole recombination leads to a more pronounced transient EPSB (Fig. 2k–m)^25,28^.

### Dynamic properties of artificial light-emitting synaptic devices as physical reservoirs

**Fig. 3:**
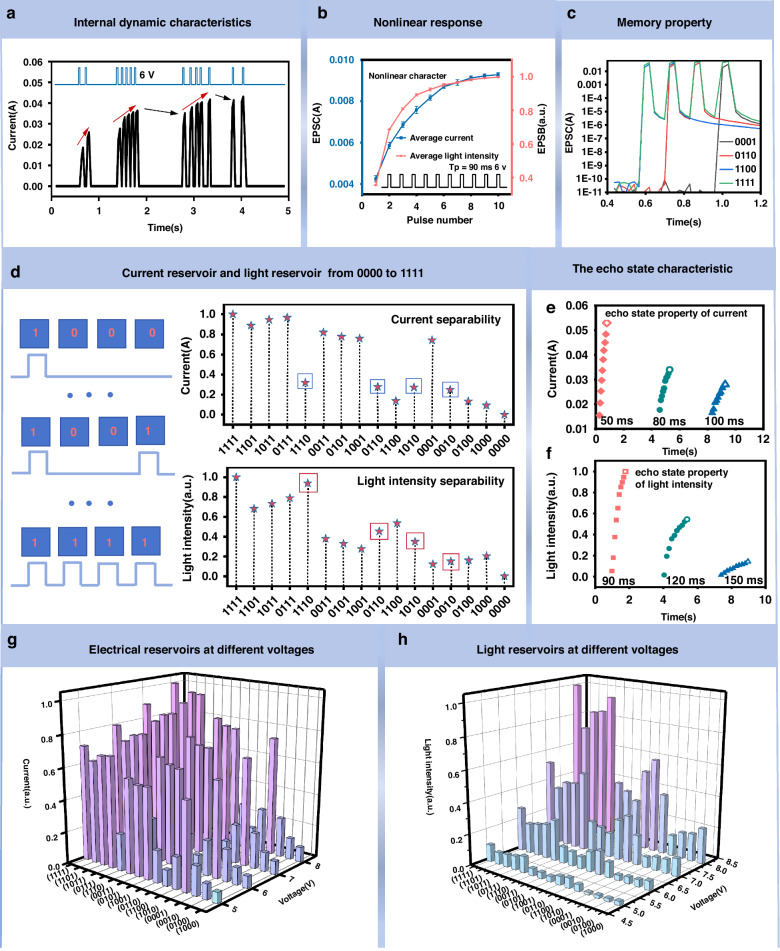
Physical reservoirs dynamic properties of artificial light-emitting synaptic devices. **a** Internal dynamic characteristics of ions: The dynamic response of the device current when stimulated by a pulse stream composed of the same electrical pulses (6 v, 90 ms) with different pulse intervals, the response trend is shown by the red arrows and black arrows. **b** Nonlinear response: Device current output and light intensity output as a function of the number of applied electrical pulses (6 v, 90 ms). **c** Short-term memory properties triggered by sequential voltage pulses. **d** Experimental read-current responses generated by sixteen 4-bit electrical pulse trains (6 v, 90 ms) ranging from (0000) to (1111). Experimental photo-responses generated by sixteen 4-bit electrical pulse trains ranging from (0000) to (1111). **e** The echo state characteristic of the device current: the current response of the device under the stimulation of 10 identical pulses (6 v, 90 ms) with different pulse intervals (50 ms, 80 ms, 100 ms). **f** The echo state characteristic of the device light intensity: the photo-response of the device under the stimulation of 10 identical pulses (6 v, 90 ms) with different pulse intervals (90 ms, 120 ms, 150 ms). **g** Current reservoir states which are generated under the stimulation of electrical pulses at different voltages (5 v, 6 v, 7 v, 8 v). **h** Light reservoir states which are generated under the stimulation of electrical pulses at different voltages (5 v, 6 v, 7 v, 8 v)

To validate the efficacy of the artificial light-emitting synaptic device as a physical reservoir for reservoir computing, an extensive investigation was conducted to analyze its dynamic characteristics. This comprehensive analysis encompassed the examination of ion dynamics^34,35^, nonlinear attenuation^36–38^, short-term memory^39–41^, separability^42–44^, and echo state characteristics^45,46^ of the device. The resulting findings unequivocally demonstrate that the device satisfies the essential criteria required for reservoir computing. Firstly, we characterize the internal dynamics of ions in artificial light-emitting synaptic devices. As shown in Fig. 3a, 14 identical electrical pulses (6 V, 30 ms) at different time intervals are applied to the device to generate the corresponding device current. When the applied electrical pulses have a short time interval, the conductance of the device exhibits a continuous increase (as shown by the red arrow in Fig. 3a). This is ascribed to the accumulation of ions near the electrode under the continuous stimulation of the electrical pulse and the continued expansion of the n and p doping regions in the device^21^ (Fig. 2h–j). When the applied electrical pulse has interval for a long time, the ions are gathered at the channel interface produce reverse migration, and the conductance of the device shows a spontaneous attenuation phenomenon^19^ (as shown by the black arrow in Fig. 3a). Figure 3b shows that under the stimulation of 10 continuous pulse sequences (6 V, 90 ms), the output current response of the artificial light-emitting synaptic device increases with the increase of the number of pulses, but the current increment gradually decreases when the number of pulses reaches a certain value. At the same time, the output light response of artificial light-emitting synaptic device also increases with the increase of pulse number, but when the pulse number reaches a certain number, the increment of light response gradually decreases. The device exhibits an obvious nonlinear current and light response to the excitation of an external electric pulse. Figure 3c shows the attenuation memory characteristics of a LES device, where the output current increases when the device is stimulated by different coding voltages and decreases over time when the voltage is withdrawn. The property of temporal short-term memory enables LES to effectively discriminate input sequences with varying temporal orders, making it an ideal choice for implementing the reservoir computing system.

Another important characteristic of a reservoir is its separability. Separability refers to the ability to distinguish inputs with different temporal characteristics using different reservoir responses. To evaluate the separability of LES, we input 16 different pulse sequences respectively (0000, 0001, 0010, 0100, 1000, 1001, 1010, 1100, 0110, 0101, 0011, 0111, 1110, 1011, 1101, 1111), “1” (6 v, 60 ms; 0 v, 90 ms), “0” (0 v, 150 ms) to LES. Before applying the pulse train, we give the LES a preset voltage pulse: “1” (6 v, 60 ms; 0 v, 90 ms). Depending on the attenuation process of the output optical signal and the electrical signal, we use different optimization methods. For the output light signal, we use optimization methods I (Fig. S11): The maximum light response produced by LES after stimulation with electric pulse sequence was taken as reservoir state. For the output electrical signal, we use optimization methods II (Fig. S11): The average of the current within the relaxation time (90 ms) after the electric pulse sequence stimulation was taken as the reservoir state. As shown in Fig. 3d and Fig. S12, 16 pulse sequences from 0000 to 1111 produce 16 clearly distinguishable electrical output states and 16 clearly distinguishable optical output states, which implies a powerful ability to map complex spatiotemporal signals to reservoir states, demonstrating excellent separability of LES. There are potential overlaps between current reservoir status distributions of four certain inputs (1110, 0110, 1010, and 0010) in the square frame of Fig. 3d, which can be further distinguished by adding light intensity reservoir states. Consequently, the feature space can streamline the classification process of the reservoir by reducing the dimensionality of the initial data from 4-bit digital inputs to 2 analog outputs, then optimizing performance through the utilization of these outputs as inputs for the linear readout layer.

Figure 3e reveals the changes in the current response of LES under three stimuli of the same amplitude of 6 v and the same pulse width of 90 ms, but with pulse intervals of 50 ms, 80 ms, and 100 ms, respectively. When the pulse interval is 50 ms, the current response increment of the device is 0.0375 A after the stimulation of 10 consecutive pulses. When the pulse interval is 80 ms, the current response increment of the device is 0.0163 A after 10 continuous pulse sequences are stimulated. When the pulse interval is 100 ms, the current response increment of the device is 0.0115 A after stimulating 10 continuous pulse sequences. This is associated with the accumulation of ions on the interface when a higher frequency electrical pulse is applied, resulting in a rapid increase in the current of the device. When a lower frequency electrical pulse is added, the large pulse interval provides sufficient time for the diffusion of ions, thus inhibiting the accumulation of ions and the continuous increase of current. Figure 3e also reveals that the current response of the device depends not only on the current electrical pulse input but also on the recent electrical pulse input. The hollow points in Fig. 3e are considered to be the final current response, and the pulse timing input of the previous period can be inferred according to the final current response, which indicates that the current response of our device has the characteristics of the echo state of the reservoir.

Figure 3f shows the changes in the light response of LES under three stimuli of the same amplitude of 6 V and the same pulse width of 90 ms, but with pulse intervals of 90 ms, 120 ms, and 150 ms, respectively. When the pulse interval time is 90 ms, the optical response increment of the device is 0.3202 V after 10 consecutive pulse stimulations. When the pulse interval time is 120 ms, the incremental optical response of the device is 0.1497 V after 10 consecutive pulses. When the pulse interval time is 150 ms, the optical response increment of the device is 0.1278 V after 10 consecutive pulses. These phenomena are ascribed to the fact that when higher frequency electrical pulses are applied, the increase in current makes the interface produce more holes and electrons, and their recombination probability greatly increases, thus enhancing the optical response of the device. When the electrical pulse of a lower frequency is applied, the increase of current is suppressed, the number of holes and electrons decreases, and their recombination probability decreases, resulting in the decreased optical response of the device. Figure 3f also indicates that the optical response of the device depends not only on the current electrical pulse input but also on the recent electrical pulse input. The hollow points in Fig. 3f are considered the final optical response, and the pulse timing input of the previous period can be inferred according to the final optical response, which indicates that the optical response of our device also has the characteristics of the echo state of the reservoir.

To demonstrate the robust capability of the LES reservoir system to map complicated spatiotemporal signals to reservoir states, we demonstrate the currents nonlinear mapping of 4-bit inputs (Fig. 3g) and the light intensity nonlinear mapping of 4-bit inputs (Fig. 3h) which are under different voltages (5 V, 6 V, 7 V, 8 V) based on the LES reservoir. The result shows the nonlinear dynamic evolution of currents and the nonlinear dynamic evolution of light intensity are both well separated. It is worth noting that the richness of nonlinear dynamic evolution in the optical reservoir is significantly higher than that of the electrical reservoir. As a result, the variation of the output light intensity can be used to map the variation of different voltage amplitudes of the pulse signal. The above data results verify that the artificial light-emitting synaptic device has the characteristics of a physical reservoir, and the reservoir system can be built based on this device. In addition, in Supplementary Information Note 2, the relationship between reservoir computing and the devices in this paper is described in detail through mathematical models.

### Realization of mixed physical node RC in the Learning of Digital Images

**Fig. 4:**
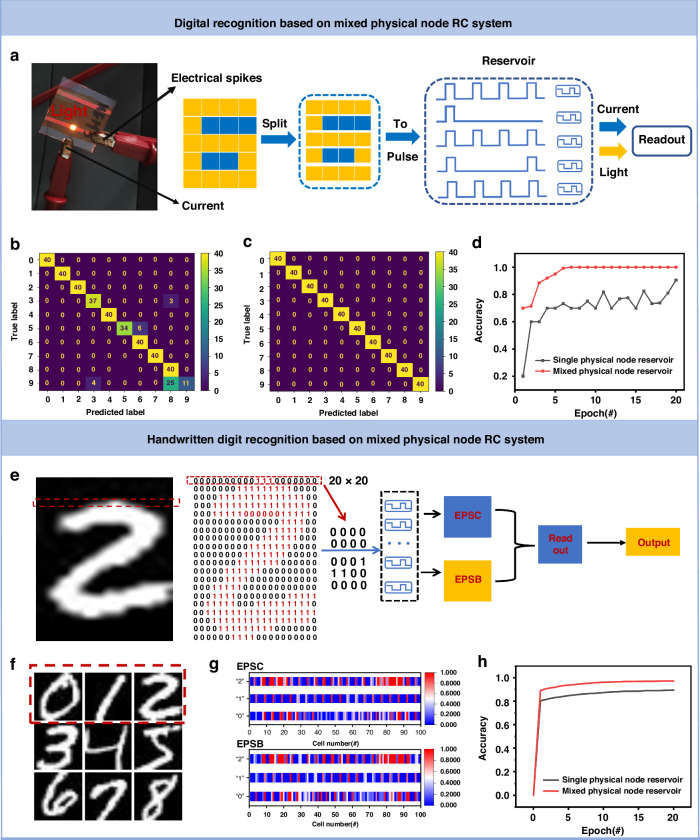
Training and performance evaluation of the digital image learning and handwritten digit image learning in mixed physical node reservoir computing system. **a** Left: Physical image of artificial light-emitting synaptic devices. Right: Schematic for the operation of photoelectric RC based on LES for classifying the digital images. Temporal sequences of pulses were applied to the five LES. **b** Confusion matrix of digital image classification after 20 training cycles (single physical node reservoir). **c** Confusion matrix of digital image classification after 20 training cycles (mixed physical node reservoir). **d** For the 20 pixels digital image, the recognition accuracy of single physical node reservoir and mixed physical node reservoir. **e** Schematic of the handwritten digit image and computing process flow. The handwritten digit image is transformed into a binary image. Next, the unused border areas were removed to reduce the original image size from 28 × 20 to 20 × 20. The binary digit image is first converted into electric pulse streams (6 v, 90 ms), and then fed to the artificial light-emitting synaptic devices-based reservoir. The recognition result is obtained after feeding the electric and optical states of the reservoir to a trained readout function. **f** Examples from the MNIST image. **g** Reservoir states (two kinds of reservoir states: electric reservoir and optical reservoir) corresponding to the three examples, showing significant differences in the reservoir states. **h** For the handwriting recognition data sets, the recognition accuracy of a single electric reservoir 89.47%, the identification accuracy of the electro-optic mixed reservoir is 97.22%

RC networks have become a strong candidate for efficient image recognition and classification due to their ability to extract high-dimensional features from spatiotemporal inputs. Compared with the single physical node RC system, the mixed physical node RC system enriches the reservoir through the two output modes of electrical response and optical response, which can effectively improve the efficiency of image recognition. To illustrate the working principle of the mixed physical node RC system, using the number “6” as an illustration. Each row of digits was converted into different coded electrical pulses. The yellow pixels correspond to the code “1” (6 v, 60 ms; 0 v, 90 ms), while the remaining blue pixels correspond to the code “0” (0 v, 150 ms). The impulses of five rows are sequentially applied to the five corresponding LES, and the resulting EPSC and EPSB were input to the readout layer for training^47,48^, as shown in Fig. 4a. With reservoir states mapped by the currents (Fig. S13) and light intensity (Fig. S14) from five LES, and the ten output neurons (labeled 0–9) correspond to the predicted numeric value of the input image. By comparing the confusion matrix obtained after 20 training cycles of the digital pictures of 20 pixels of the single physical node reservoir and the mixed physical node reservoir (Figs. S15 and S16), the mixed physical node reservoir can recognize all the digits 100% accurately, while the single physical node reservoir cannot recognize the number “3”, “5”, “8” and “9” accurately (Fig. 4b and c). As shown in Fig. 4d, the mixed physical node RC system can accurately identify every temporal sequence of pulses from the 10 original images after 6 training epochs, with a matching accuracy of 100%.

To further demonstrate the advantage of mixed physical node reservoirs, more sophisticated handwritten digit recognition is executed. For handwritten digital image recognition, the image is first converted into a binary pixel image, as shown in Fig. 4e. In theory, however, using the whole row as a single stream of input pulses produces 2^20^ different patterns, which is difficult for LES to distinguish. To solve this problem, each row is divided into 5 sections, each containing 4 pixels to separate the input more efficiently. Using the picture of the number 2 as an illustration, a row (marked by a red line) is divided into five parts: 0000, 0000, 0001, 1100, 0000 (Fig. 4e). The binary image is converted into the corresponding voltage pulse sequence, which is input into the mixed physical node reservoir, so as to generate 200 kinds of photoelectric signal mixed reservoir state. The simulated reservoir states shown in Fig. 4g correspond to the three handwritten digital pictures (highlighted by the red line in Fig. 4f), demonstrating the significant difference in the LES reservoir states. Finally, the reservoir states are trained in the readout network (see experiment section and Supplementary Information Note 1 for details). The recognition accuracy of the handwritten digital images is shown in Fig. 4h. After 20 training sessions, the recognition accuracy of the mixed physical node RC system achieved 97.22%, while the single physical node was only 89.47%.

### Realization of mixed physical node RC for multichannel image learning

**Fig. 5:**
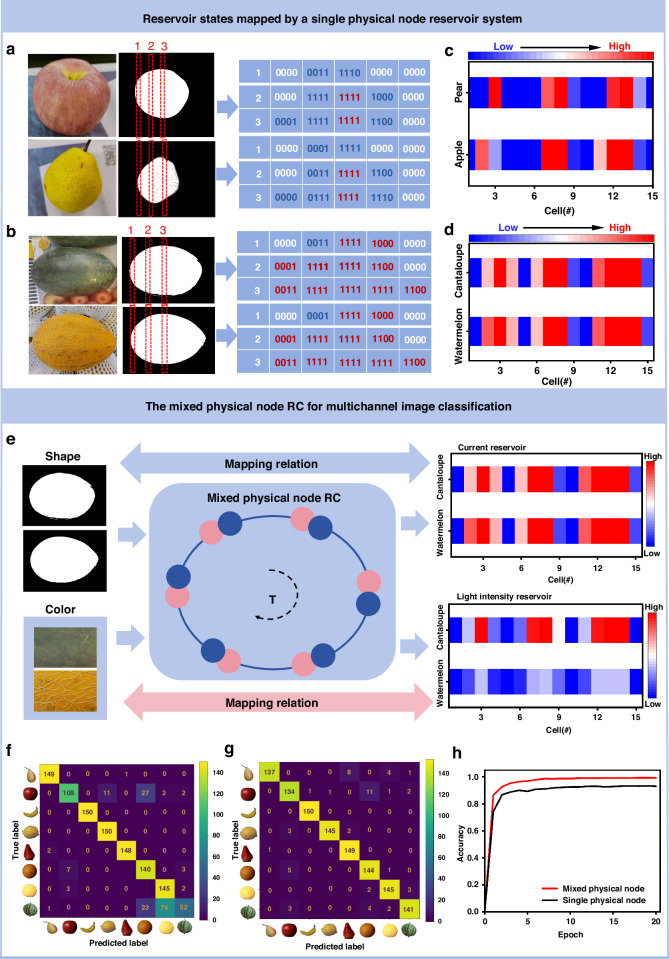
Training and recognition of the multichannel image learning in mixed physical node reservoir computing system. **a** Image preprocessing for fruit images with different shapes: After image processing, the images of apple and pear are converted into voltage pulse code. **b** Image preprocessing for fruit images with similar shapes: After image processing, the images of watermelon and cantaloupe are converted into voltage pulse code. **c** Current reservoir states of apple and pear mapped through the single physical node reservoir system. **d** Current reservoir states of watermelon and cantaloupe mapped through the single physical node reservoir system. **e** A schematic of multi-dimensional feature recognition of fruit images based on mixed physical node system. **f** Fruit image confusion matrix recognized by the single electric node reservoir. **g** Fruit image confusion matrix recognized by the mixed physical node reservoir. **h** For the multichannel fruit image dataset, the recognition accuracy of a single electric reservoir 93.16%, and the identification accuracy of the electro-optic mixed reservoir is 99.25%

The reservoir system based on memristor mostly uses binary dataset for coding. This binary coding scheme encodes the shape of the identified object and inputs it into the reservoir as a pulse sequence to generate the corresponding current reservoir state. This can reduce the difficulty of signal processing of memristor to some extent. However, this kind of binary coding which recognizes the task only by mapping the shape of an object will inevitably cause data distortion and recognition errors to a certain extent. For instance, in Fig. 5a, after preprocessing the images of two kinds of fruits, apple, and pear, converting the images into binary pixel maps, and then constructing their respective reservoir reservoirs according to different codes, it can be seen that there are large differences in their reservoirs (Fig. 5c), and accurate identification can be achieved (Fig. S17a). However, for watermelons and cantaloupes with small shape differences (Fig. 5b), the two reservoirs constructed by the memristor-based RC system have high similarity (Fig. 5d), resulting in the misidentification of the two fruits (Fig. S17b). In Fig. 5e and Fig. S17c, the mixed physical node RC system in this paper uses the two outputs of reading current and light intensity respectively to construct a dual-modal reservoir for mapping the shape and gray value of task objects, current reservoirs, and light intensity reservoirs can be obtained (the right part of Fig. 5e). The current reservoir state can distinguish pulse sequences of different time series input by its short-term memory characteristics (Fig. 3c), and the light intensity reservoir state can distinguish pulse sequences of different amplitude by its dynamic emission characteristics (Fig. 3h). When current reservoirs are similar in height, objects can be distinguished by light intensity reservoirs. To validate the ability of nonlinear mapping input information on the mixed physical node reservoir system, we perform image recognition of the multichannel fruit dataset with the mixed physical node reservoir system (Figs. S18 and S19): By linearly mapping grayscale values [0, 255] to voltage amplitude values [4 V, 10 V] (Fig. S20), the gray value is calculated by the formula:

3 $${Gray}=\root{2.2}\of{\frac{{R}^{2.2}+{(1.5G)}^{2.2}+{(0.6B)}^{2.2}}{1+{1.5}^{2.2}+{0.6}^{2.2}}}$$

Then, converting the images into continuous voltage streams that can be processed by LES to generate corresponding current and light intensity reservoir state. Finally, the reservoir states are trained in the readout network (see experiment section for details). By comparing the confusion matrix of single physical node reservoirs (Fig. 5f) and mixed physical node reservoirs (Fig. 5g) after 20 training cycles, the mixed physical node reservoir can identify the target more accurately. The recognition accuracy of the multichannel fruit dataset is shown in Fig. 5h. The final recognition rate is improved to 99.25% compared to 93.16% for a single physical node reservoir.

Finally, we summarize the advantages of the mixed physical node reservoir system in the following aspects: (i) Mixed reservoir nodes: Unlike traditional dynamic memristors for RC, this device not only maps timing signals through dynamic current changes, but also extracts spatiotemporal and amplitude characteristics of signals in parallel through dynamic luminescence. As a result, the device as a reservoir layer can map timing signals in multiple physical dimensions, significantly improving the richness of reservoir states. (ii) Visual information presentation: The device can directly map different timing signals in the form of dynamic emission, directly avoiding the crosstalk problem with the dynamic current reservoir state while visualizing, which is the main challenge of traditional hardware reservoir devices. (iii) Multi-feature fusion task recognition: Compared with the evolution of timing signals by a single reservoir node, the reservoir system of mixed physical nodes greatly expands the extraction range of spatiotemporal features of the input signal by means of the parallel mapping of memory current and dynamic luminescence, and the multichannel image recognition task accuracy is improved from 93.16% to 99.25%.

## Discussion

In conclusion, we propose a mixed physical node reservoir system. This system utilizes the ion-electron coupling principle of artificial light-emitting synaptic devices to generate optical output with nonlinear optical effects and electrical output with memory characteristics. By extracting these characteristic outputs, a mixed physical node reservoir can be constructed. On this basis, the handwriting digit recognition accuracy is higher than the single-mode reservoir system. In addition, different from the previous single physical node memristor reservoir system, the mixed physical node reservoir system maps the shape of the image to the current reservoir and the gray value to the light intensity reservoir through the characteristics of dual eigenvalue mapping, which can realize the recognition of RGB multichannel image, and can be applied to more scenarios and more complex computing tasks. Hence, this innovative reservoir system with a parallel output of optoelectronic signals shows great potential in next-generation optoelectronic mixed neural networks and material-algorithm collaborative design.

## Materials and methods

### Materials

LiCF_3_SO_3_ (12 mg) and PEO (8 mg) were dissolved in 1 mL of cyclohexanone, respectively, and then magnetically stirred for 18 h at 50 °C in an ambient atmosphere. MEH-PPV (12 mg) was dissolved in 1 mL trichloromethane and then annealed at 50 °C for 10 min. The three solutions were mixed in a mass ratio of MEH-PPV: PEO: LiCF_3_SO_3_ = 1:0.25:0.04. MXene solution of 5 mg/mL was mixed with the above solution in a ratio of 0.5:100. Then the mixing solution was magnetically stirred for 3 h at 50 °C in an ambient atmosphere to make the active layer.

### Fabrication of LES

The active layer was prepared by spin coating the mixed solution onto clean ITO electrodes-coated glass substrates at 1400 rpm for 40 s, and then annealed at 90 °C for 10 min in a nitrogen glove box. Ag electrodes of 50 nm were deposited onto the active layer by thermal evaporation using a shadow mask.

### Optoelectronic measurement

The electrical performance was characterized by the semiconductor parameter analyzer (Keysight B2902A). Light signals were detected by a non-amplified Photodetector (ET-2030) and converted to readable value by an oscilloscope (Keysight DSOX1202). The EL spectra of the devices were obtained using FL 4600.

### Network training

A supervised learning algorithm was adopted to train the readout layer for image recognition tasks (Figs. 4 and 5). The reservoir states, which are represented by the current response and light intensity response of light-emitting synaptic devices, are fed to the readout network. RC readout networks adopt the cross-entropy loss function to calculate the loss. The back-propagation (BP) and batch gradient descent algorithms were used to update the weights and minimize the loss value using the SGD optimizer. Then, the ReLU regression was used to fit the weight of readout, to get the maximum probability index. Specifically, the readout network of size 5 × 10 and 10 × 10 for simple digital image recognition tasks used a learning rate of 0.05, and a batch size of 16. The accuracy of the handwritten digital images classification, shown in Fig. 4e, was obtained using another readout network of size 100 × 100 × 10 and 200 × 100 × 10, where the learning rate was 0.001, and the batch size was 32. And using another readout network of size 2500 × 1024 × 8 and 5000 × 1024 × 8 for the recognition task of multichannel image, where the learning rate = 0.000005 and batch size = 64.

### Supplementary information

SUPPLEMENTAL MATERIAL for Towards mixed physical node reservoir computing: Light-emitting synaptic reservoir system with dual photoelectric output

## References

[1] Hopfield, J. J. Neural networks and physical systems with emergent collective computational abilities. *Proc. Natl Acad. Sci. USA* **79**, 2554–2558 (1982). 6953413 10.1073/pnas.79.8.2554 PMC346238

[2] LeCun, Y., Bengio, Y. & Hinton, G. Deep learning. *Nature* **521**, 436–444 (2015). 26017442 10.1038/nature14539

[3] Yang, J. Q. et al. Neuromorphic engineering: from biological to spike-based hardware nervous systems. *Adv. Mater.* **32**, 2003610 (2020). 10.1002/adma.202003610 33165986

[4] Wei, Z. M. Reservoir computing with 2D materials. *Nat. Electron.* **5**, 715–716 (2022). 10.1038/s41928-022-00872-1

[5] Sun, Y. M. et al. Experimental demonstration of a skyrmion-enhanced strain-mediated physical reservoir computing system. *Nat. Commun.* **14**, 3434 (2023). 37301906 10.1038/s41467-023-39207-9 PMC10257712

[6] Nakajima, K. Physical reservoir computing—an introductory perspective. *Jpn. J. Appl. Phys.* **59**, 060501 (2020). 10.35848/1347-4065/ab8d4f

[7] Qi, Z. Y. et al. Physical reservoir computing based on nanoscale materials and devices. *Adv. Funct. Mater.* **33**, 2306149 (2023). 10.1002/adfm.202306149

[8] Du, C. et al. Reservoir computing using dynamic memristors for temporal information processing. *Nat. Commun.* **8**, 2204 (2017). 29259188 10.1038/s41467-017-02337-y PMC5736649

[9] Sillin, H. O. et al. A theoretical and experimental study of neuromorphic atomic switch networks for reservoir computing. *Nanotechnology* **24**, 384004 (2013). 23999129 10.1088/0957-4484/24/38/384004

[10] Vandoorne, K. et al. Experimental demonstration of reservoir computing on a silicon photonics chip. *Nat. Commun.* **5**, 3541 (2014). 24662967 10.1038/ncomms4541

[11] Torrejon, J. et al. Neuromorphic computing with nanoscale spintronic oscillators. *Nature* **547**, 428–431 (2017). 28748930 10.1038/nature23011 PMC5575904

[12] Sun, L. F. et al. In-sensor reservoir computing for language learning via two-dimensional memristors. *Sci. Adv.* **7**, eabg1455 (2021). 33990331 10.1126/sciadv.abg1455 PMC8121431

[13] Park, S. O. et al. Experimental demonstration of highly reliable dynamic memristor for artificial neuron and neuromorphic computing. *Nat. Commun.* **13**, 2888 (2022). 35660724 10.1038/s41467-022-30539-6 PMC9166790

[14] Ilyas, N. et al. Analog switching and artificial synaptic behavior of Ag/SiO_*x*_:Ag/TiO_*x*_/p^++^-Si memristor device. *Nanoscale Res. Lett.* **15**, 30 (2020). 32006131 10.1186/s11671-020-3249-7 PMC6994582

[15] Zhang, Z. F. et al. In-sensor reservoir computing system for latent fingerprint recognition with deep ultraviolet photo-synapses and memristor array. *Nat. Commun.* **13**, 6590 (2022). 36329017 10.1038/s41467-022-34230-8 PMC9633641

[16] Zha, J. J. et al. Electronic/optoelectronic memory device enabled by tellurium-based 2D van der Waals heterostructure for in-sensor reservoir computing at the optical communication band. *Adv. Mater.* **35**, 2211598 (2023). 10.1002/adma.202211598 36857506

[17] Wang, M. et al. Gesture recognition using a bioinspired learning architecture that integrates visual data with somatosensory data from stretchable sensors. *Nat. Electron.* **3**, 563–570 (2020). 10.1038/s41928-020-0422-z

[18] Moon, J. et al. Temporal data classification and forecasting using a memristor-based reservoir computing system. *Nat. Electron.* **2**, 480–487 (2019). 10.1038/s41928-019-0313-3

[19] Zhang, G. H. et al. Functional materials for memristor-based reservoir computing: dynamics and applications. *Adv. Funct. Mater.* **33**, 2302929 (2023). 10.1002/adfm.202302929

[20] Jaafar, A. H. et al. 3D-structured mesoporous silica memristors for neuromorphic switching and reservoir computing. *Nanoscale* **14**, 17170–17181 (2022). 36380717 10.1039/D2NR05012A

[21] Yamaguchi, H. Efficient encoding of colored pictures in R, G, B components. *IEEE Trans. Commun.* **32**, 1201–1209 (1984). 10.1109/TCOM.1984.1095992

[22] Liu, J. et al. Lithium ion batteries: uniform hierarchical Fe3O4@polypyrrole nanocages for superior lithium ion battery anodes. *Adv. Energy Mater.* **6**, 1600256 (2016). 10.1002/aenm.201600256

[23] Nazeeruddin, M. K. et al. Efficient green-blue-light-emitting cationic iridium complex for light-emitting electrochemical cells. *Inorg. Chem.* **45**, 9245–9250 (2006). 17083222 10.1021/ic060495e

[24] Zhu, M. P., Yuan, X. T. & Ni, G. Magneto-electroluminescence in ITO/MEH-PPV:PEO:LiCF_3_SO_3_/Al polymer light-emitting electrochemical cells. *Micromachines* **10**, 546 (2019). 31426537 10.3390/mi10080546 PMC6723417

[25] Chee, K. J. et al. Polymer light-emitting electrochemical cell blends based on selection of lithium salts, LiX [X = trifluoromethanesulfonate, hexafluorophosphate, and bis(trifluoromethylsulfonyl)imide] with low turn-on voltage. *J. Phys. Chem. C* **120**, 11324–11330 (2016). 10.1021/acs.jpcc.6b00989

[26] Zhu, X. T. et al. Negative phototransistors with ultrahigh sensitivity and weak-light detection based on 1D/2D molecular crystal p–n heterojunctions and their application in light encoders. *Adv. Mater.* **34**, 2201364 (2022). 10.1002/adma.202201364 35324012

[27] Zeng, H. A. et al. A light-emitting electrochemical artificial synapse with dual output of photoelectric signals. *Sci. China Mater.* **65**, 2511–2520 (2022). 10.1007/s40843-021-2029-y

[28] Cao, Y. et al. Efficient, fast response light-emitting electrochemical cells: electroluminescent and solid electrolyte polymers with interpenetrating network morphology. *Appl. Phys. Lett.* **68**, 3218–3220 (1996). 10.1063/1.116442

[29] Li, E. L. et al. MXene based saturation organic vertical photoelectric transistors with low subthreshold swing. *Nat. Commun.* **13**, 2898 (2022). 35610215 10.1038/s41467-022-30527-w PMC9130145

[30] Shi, Y. Z. et al. MXene-based mesoporous nanosheets toward superior lithium ion conductors. *Adv. Energy Mater.* **10**, 1903534 (2020). 10.1002/aenm.201903534

[31] Liu, Y. Q. et al. A one-structure-layer PDMS/Mxenes based stretchable triboelectric nanogenerator for simultaneously harvesting mechanical and light energy. *Nano Energy* **86**, 106118 (2021). 10.1016/j.nanoen.2021.106118

[32] Pan, Q. W. et al. 2D MXene-containing polymer electrolytes for all-solid-state lithium metal batteries. *Nanoscale Adv.* **1**, 395–402 (2019). 36132461 10.1039/C8NA00206A PMC9473207

[33] Tang, W. J. et al. Simultaneously enhancing the thermal stability, mechanical modulus, and electrochemical performance of solid polymer electrolytes by incorporating 2D sheets. *Adv. Energy Mater.* **8**, 1800866 (2018). 10.1002/aenm.201800866

[34] Liang, X. C. et al. Multimode transistors and neural networks based on ion-dynamic capacitance. *Nat. Electron.* **5**, 859–869 (2022). 10.1038/s41928-022-00876-x

[35] Wang, Y. Y. et al. Self-doping memristors with equivalently synaptic ion dynamics for neuromorphic computing. *ACS Appl. Mater. Interfaces* **11**, 24230–24240 (2019). 31119929 10.1021/acsami.9b04901

[36] Kim, G. et al. Retention secured nonlinear and self-rectifying analog charge trap memristor for energy-efficient neuromorphic hardware. *Adv. Sci.* **10**, 2205654 (2023). 10.1002/advs.202205654 PMC9875615 36437042

[37] Ghosh, P. K. et al. CMOS-based memristor emulator circuits for low-power edge-computing applications. *Electronics* **12**, 1654 (2023). 10.3390/electronics12071654

[38] Jo, S. et al. Memristor neural network training with clock synchronous neuromorphic system. *Micromachines* **10**, 384 (2019). 31181763 10.3390/mi10060384 PMC6632029

[39] Rao, J. et al. An electroforming-free, analog interface-type memristor based on a SrFeOx epitaxial heterojunction for neuromorphic computing. *Mater. Today Phys.* **18**, 100392 (2021). 10.1016/j.mtphys.2021.100392

[40] Zhang, X. M. et al. Emulating short-term and long-term plasticity of bio-synapse based on Cu/a-Si/Pt memristor. *IEEE Electron Device Lett.* **38**, 1208–1211 (2017). 10.1109/LED.2017.2722463

[41] Wang, Z. R. et al. Resistive switching materials for information processing. *Nat. Rev. Mater.* **5**, 173–195 (2020). 10.1038/s41578-019-0159-3

[42] Leng, Y. B. et al. Recent progress in multiterminal memristors for neuromorphic applications. *Adv. Electron. Mater.* **9**, 2300108 (2023). 10.1002/aelm.202300108

[43] Snyder, D., Goudarzi, A. & Teuscher, C. Computational capabilities of random automata networks for reservoir computing. *Phys. Rev. E* **87**, 042808 (2013). 10.1103/PhysRevE.87.042808 23679474

[44] Dale, M. et al. A substrate-independent framework to characterize reservoir computers. *Proc. R. Soc. A Math. Phys. Eng. Sci.* **475**, 20180723 (2019). 10.1098/rspa.2018.0723 PMC6598063 31293353

[45] Sayyaparaju, S. et al. Circuit techniques for efficient implementation of memristor based reservoir computing. *In Proc. 2020 IEEE International Symposium on Circuits and Systems* 1–5 (IEEE, Seville, Spain, 2020).

[46] Lukoševičius, M. & Jaeger, H. Reservoir computing approaches to recurrent neural network training. *Comput. Sci. Rev.* **3**, 127–149 (2009). 10.1016/j.cosrev.2009.03.005

[47] Wang, R. et al. Deep reservoir computing based on self-rectifying memristor synapse for time series prediction. *Appl. Phys. Lett.* **123**, 042109 (2023). 10.1063/5.0158076

[48] Wang, R. et al. Bio-inspired in-sensor compression and computing based on phototransistors. *Small* **18**, 2201111 (2022). 10.1002/smll.202201111 35534444

