# Supplementary material for: Towards mixed physical node reservoir computing: light-emitting synaptic reservoir system with dual photoelectric output
Source: Light Sci Appl. 2024 Aug 1;13:179. doi: 10.1038/s41377-024-01516-z (PMC11291830; doi:10.1038/s41377-024-01516-z)
Supplement: Supplementary file 1 — SUPPLEMENTAL MATERIAL for Towards mixed physical node reservoir computing: Light-emitting synaptic reservoir system with dual photoelectric output [file 41377_2024_1516_MOESM1_ESM.docx]

Supplementary Materials for

**Towards mixed physical node reservoir computing: Light-emitting synaptic reservoir system with dual photoelectric output**

Minrui Lian^1,2,3,6^, Changsong Gao^1,2,6^ Zhenyuan Lin^1,2,3^, Liuting Shan^1,2^, Cong Chen^1,2^, Yi Zou^1,2^, Enping Cheng^1,2^, Changfei Liu^1,2,3^, Tailiang Guo^1,2^, Wei Chen^3,4,5^ and Huipeng Chen*^,1,2^

^1^Institute of Optoelectronic Display, National & Local United Engineering Lab of Flat Panel Display Technology, Fuzhou University, Fuzhou 350002, China, E-mail: hpchen@fzu.edu.cn

^2^Fujian Science & Technology Innovation Laboratory for Optoelectronic Information of China, Fuzhou 350100, China

^3^Joint School of National University of Singapore and Tianjin University, International Campus of Tianjin University, Fuzhou 350207

^4^Department of Chemistry, National University of Singapore, 3 Science Drive 3, Singapore, 117543.

^5^Department of Physics, National University of Singapore, 3 Science Drive 3, Singapore, 117543.

^6^ These authors contributed equally: Minrui Lian, Changsong Gao.

*****Corresponding author

Email addresses: [hpchen@fzu.edu.cn](mailto:hpchen@fzu.edu.cn)

**Supplementary Figures**


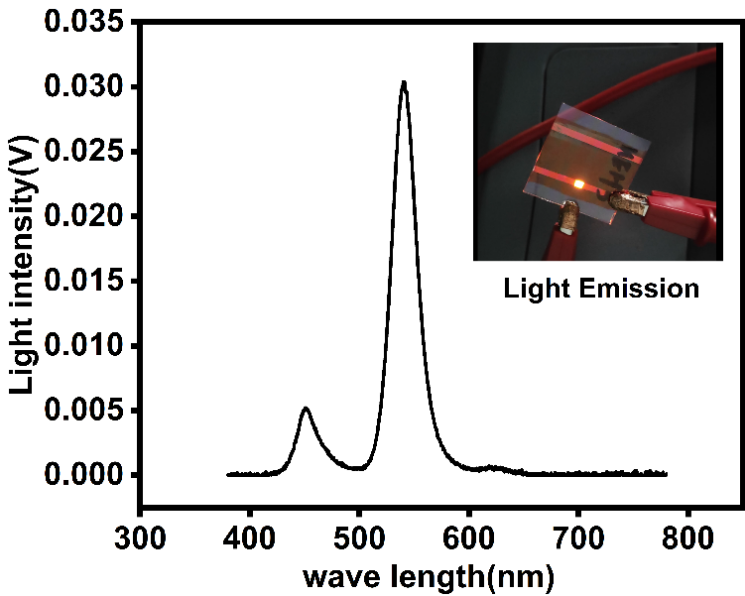


**Fig.S1. EL spectrum of LES.** The EL peak is at the wavelength of ~ 540 nm. (the inset is an optical photograph of the working LES)


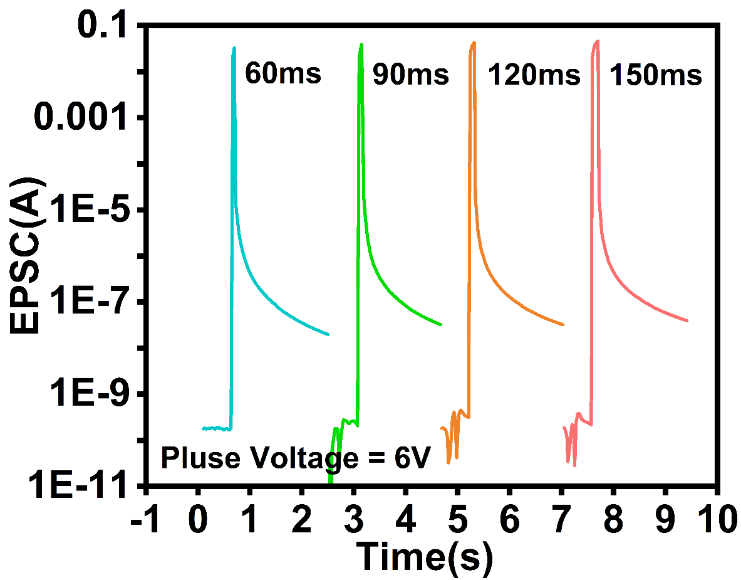


**Fig.S2. Spike duration time-dependent plasticity (SDDP) with pulse width from 60 ms to 150 ms (V pre = 6 V).**

**
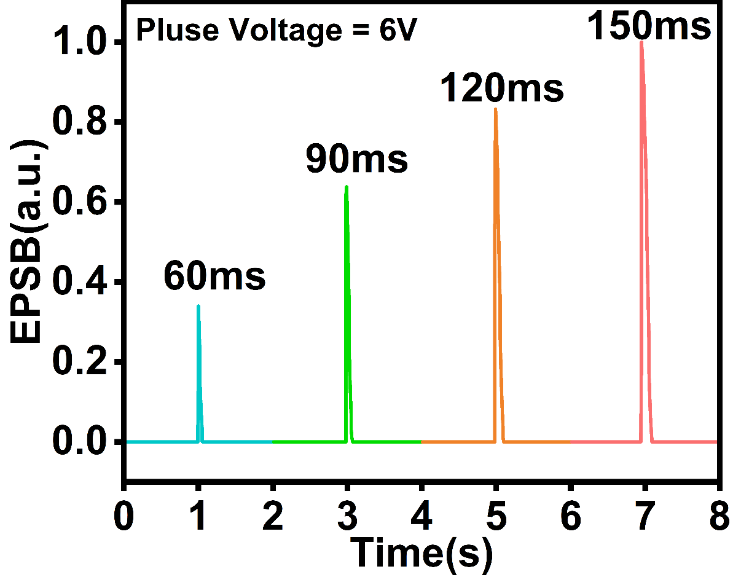
**

**Fig.S3. EPSB induced by presynaptic spikes with different duration time (60 ms−150 ms).**


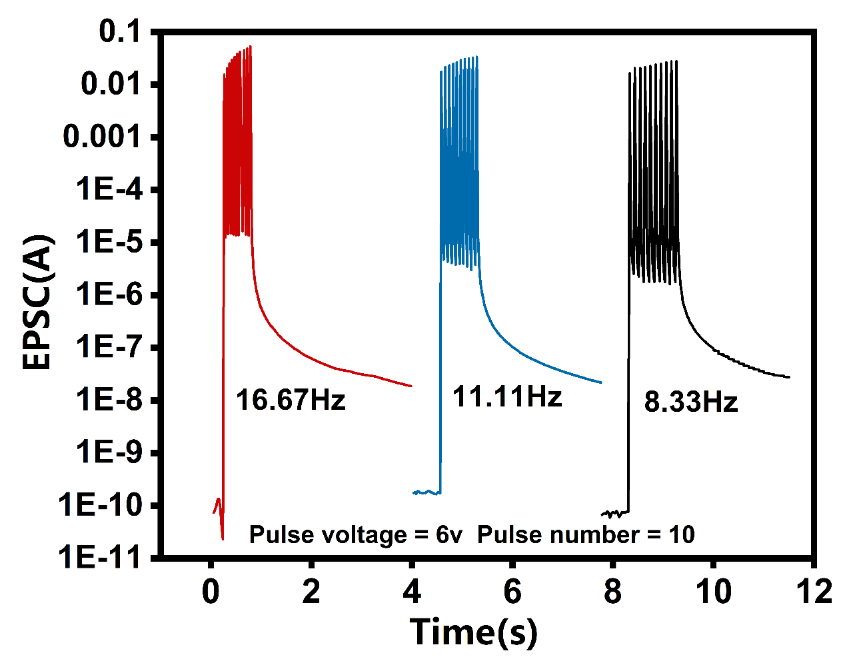


**Fig.S4. Spike rate dependent plasticity (SRDP) with pulse frequency from 8.33 Hz to 16.67 Hz (V pre = 6 V, 60 ms).**


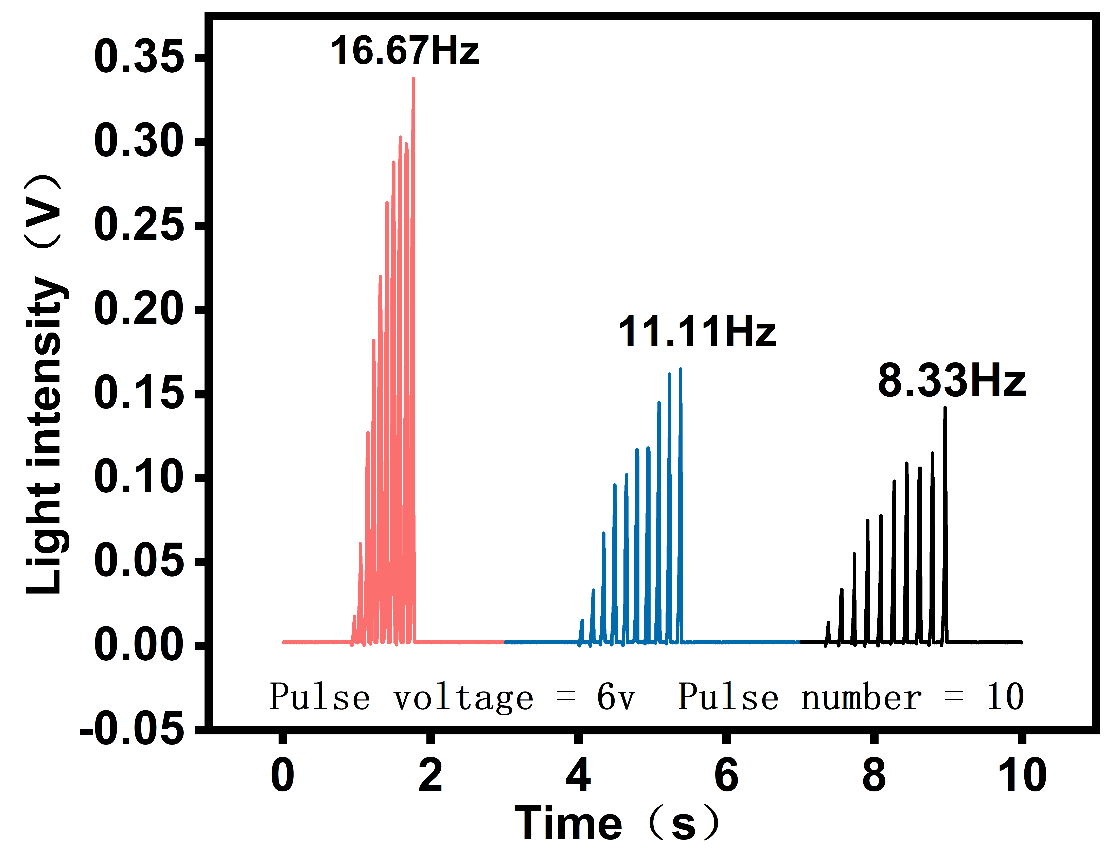


**Fig.S5. EPSB triggered by presynaptic spikes with pulse frequency from 8.33 Hz to 16.67 Hz.**


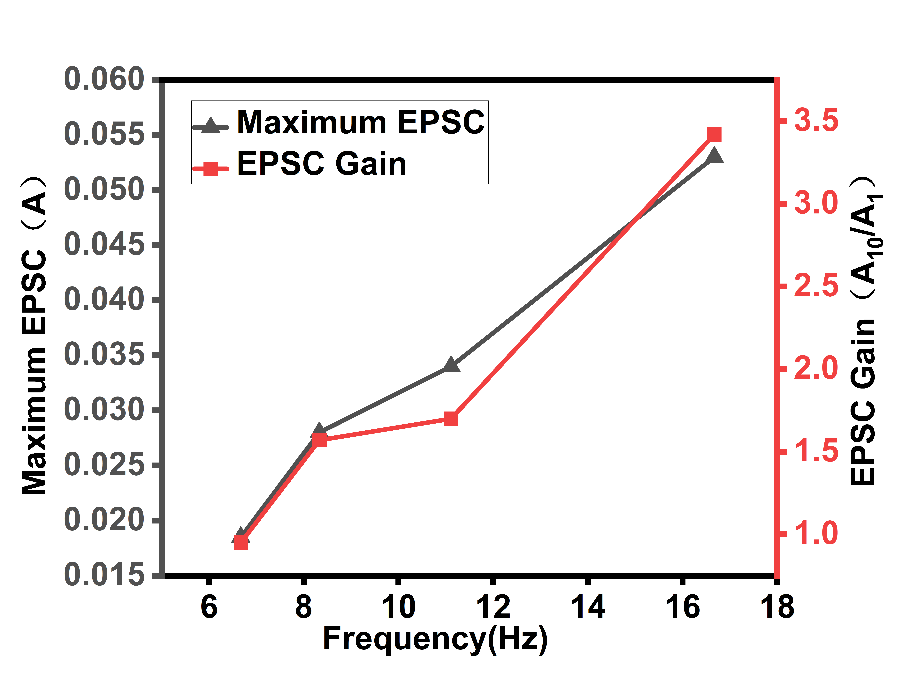


**Fig.S6. Maximum EPSC and EPSC gain (A_10_/A_1_) (determined by the ratio of the 10th EPSC peak (A_10_) to the first EPSC peak (A_1_)) of the artificial synapse from 8.33 Hz to 16.67 Hz (6 V amplitude, 60ms duration). The maximum EPSC value increased steadily from 0.0185 A to 0.053 A. The EPSC gain in different frequencies was measured and gradually increased from 0.95 to 3.419.**

**
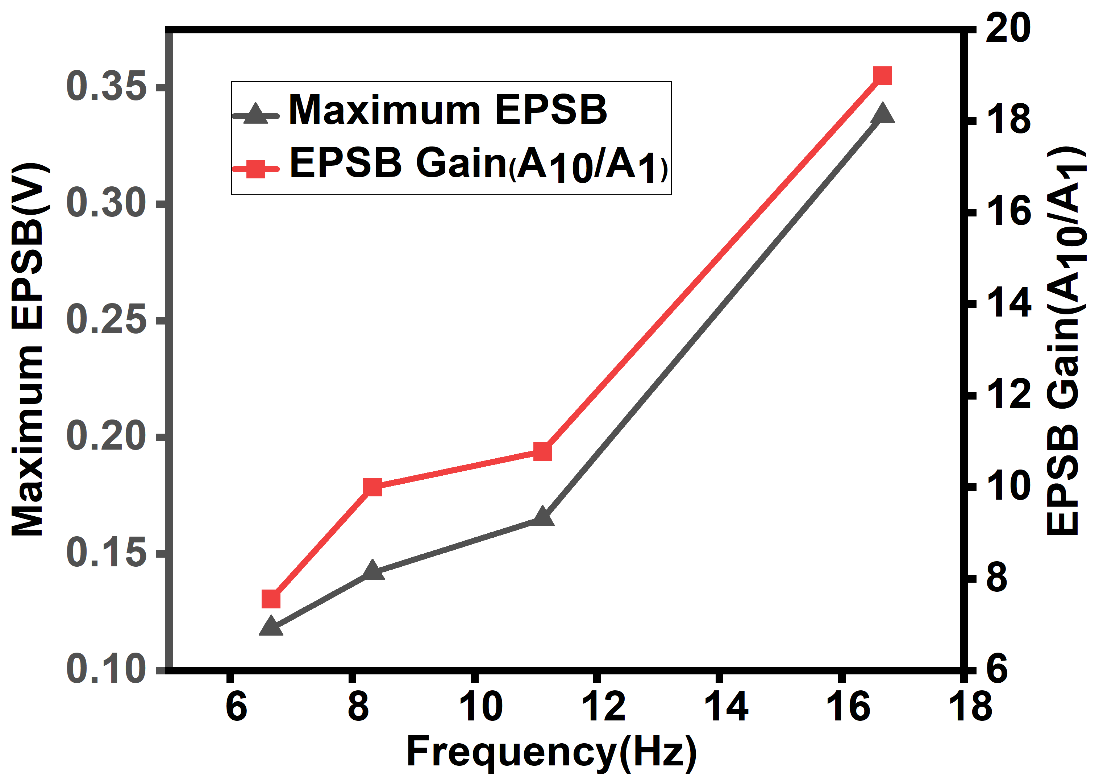
**

**Fig.S7. Maximum EPSB and EPSB gain (A_10_/A_1_) (determined by the ratio of the 10th EPSB peak (A_10_) to the first EPSB peak (A_1_)) of the artificial synapse from 8.33 Hz to 16.67 Hz (6 V amplitude, 60ms duration). The maximum EPSB value increased steadily from 0.0118 V to 0.338 V. The EPSC gain in different frequencies was measured and gradually increased from 7.56 to 18.99.**

**
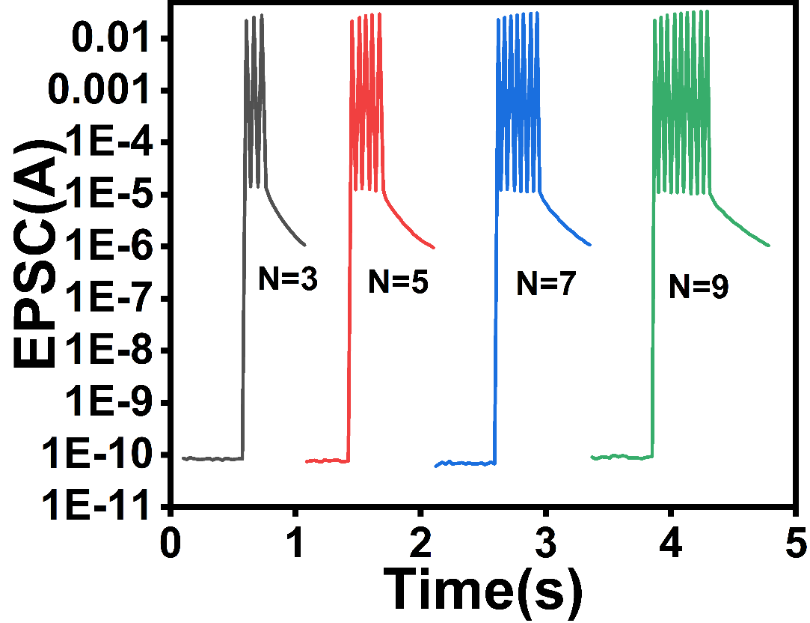
**

**Fig.S8. Spike number-dependent plasticity (SNDP) with spike number from N=3 to N=9 (6 V amplitude, 30 ms duration).**

**
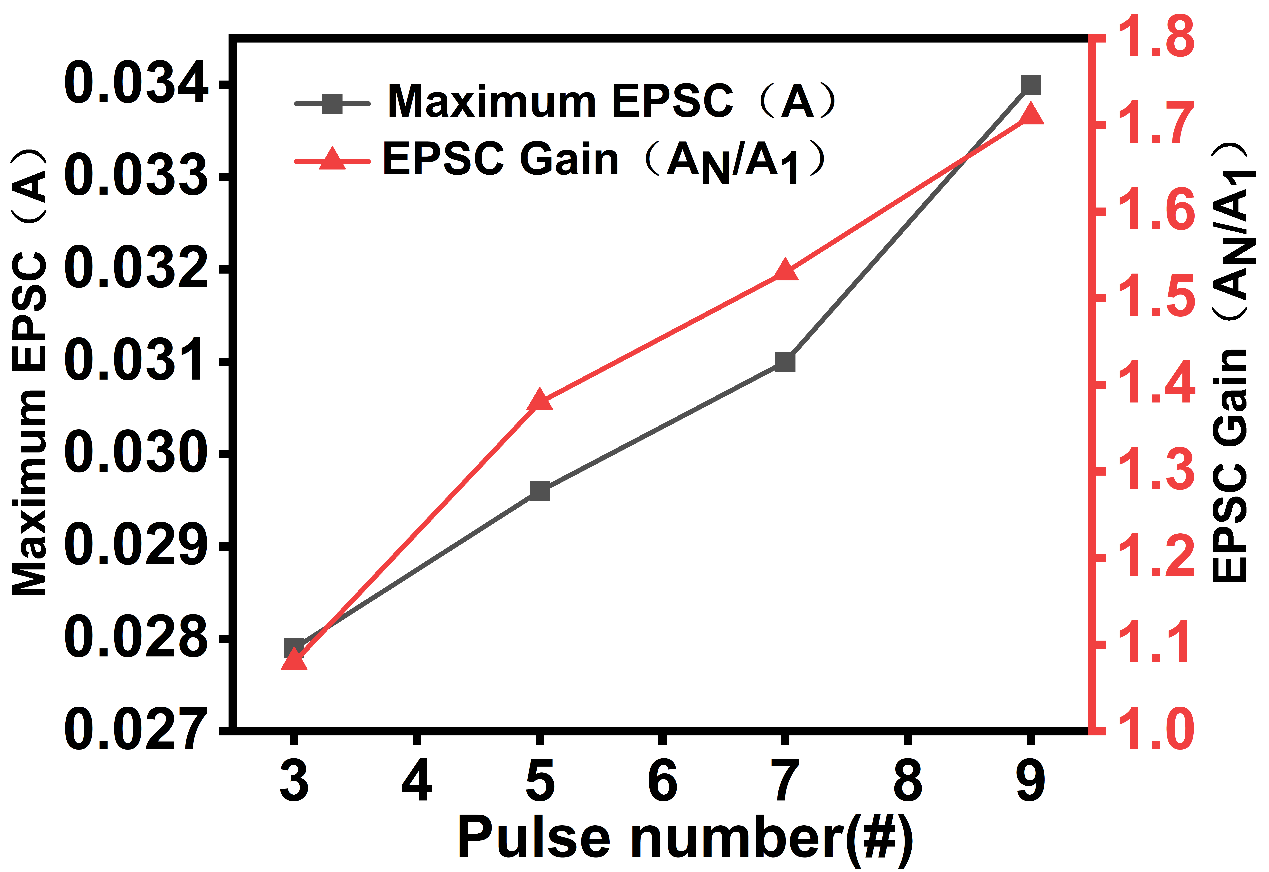
**

**Fig.S9. Maximum EPSCs and EPSC gain (A_N_/A_1_) of artificial synapse from 3 pulses to 9 pulses (6 V amplitude, 30 ms duration). With the increase of spike number from 3 to 9, the maximum EPSC increased steadily from 0.0279 A to 0.034 A. EPSC gain A_N_/A_1_ (determined by the ratio of the last EPSC peak (A_N_) to the first EPSC peak (A_1_)) increased from 1.08 to 1.71.**

**
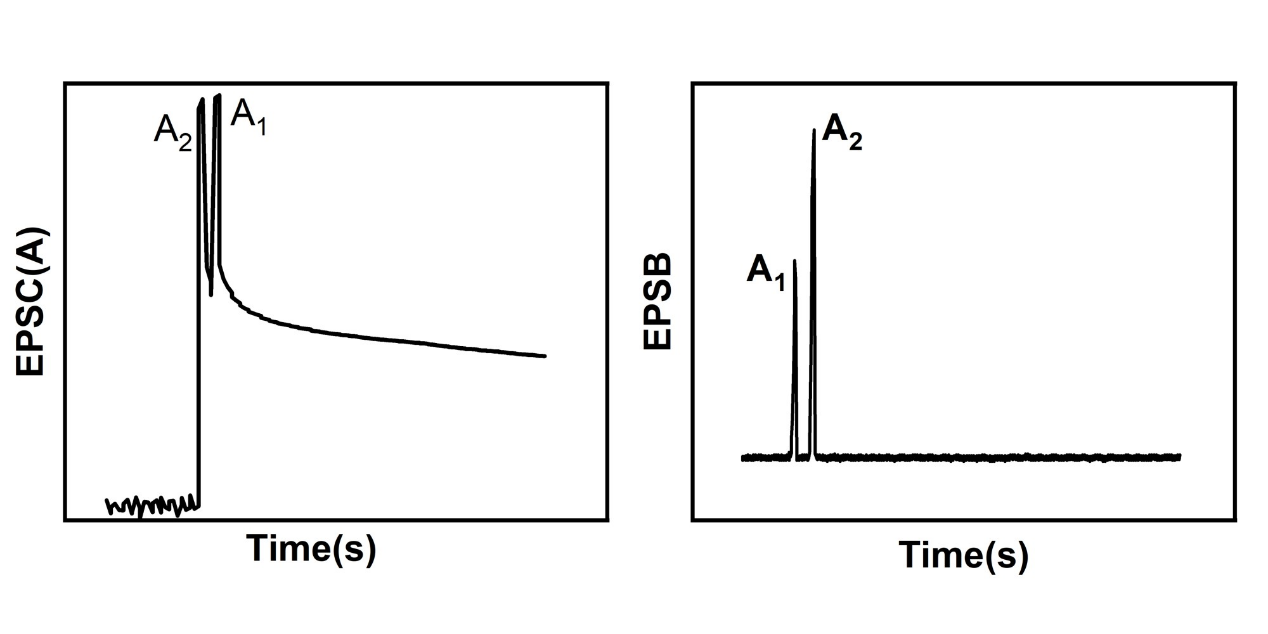
**

**Fig.S10. The PPF behavior simulated by the artificial light-emitting synaptic device.**

**
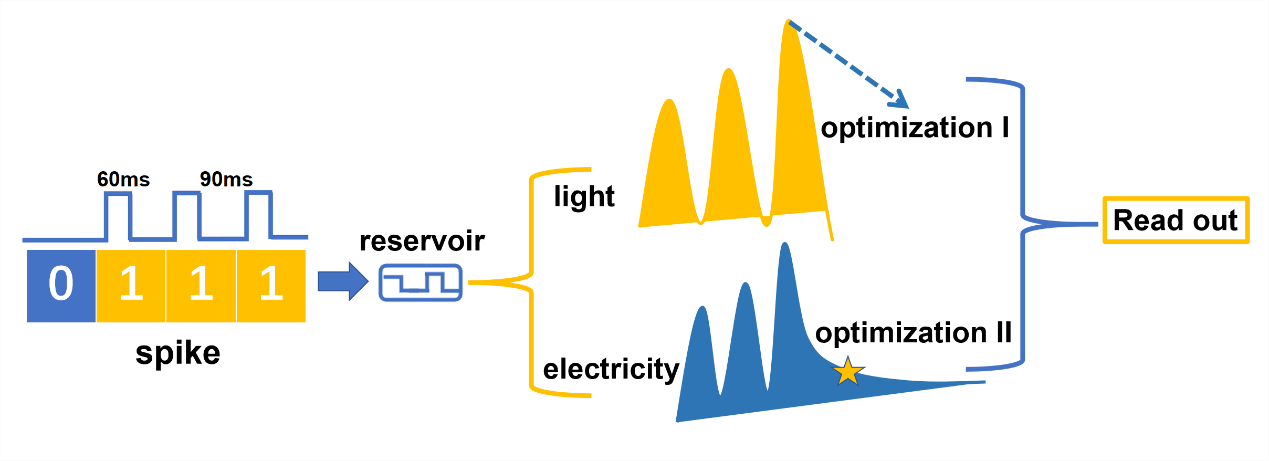
**

**Fig.S11. Two different signal optimization methods for output optical signal and electrical signal.** For the output light signal, we use sampling method I: The maximum light response produced by LES after stimulation with electric pulse sequence was taken as reservoir state. For the output electrical signal, we use sampling method II: The average of the current within the relaxation time (90 ms) after the stimulation of the electrical pulse sequence is taken as the reservoir state.


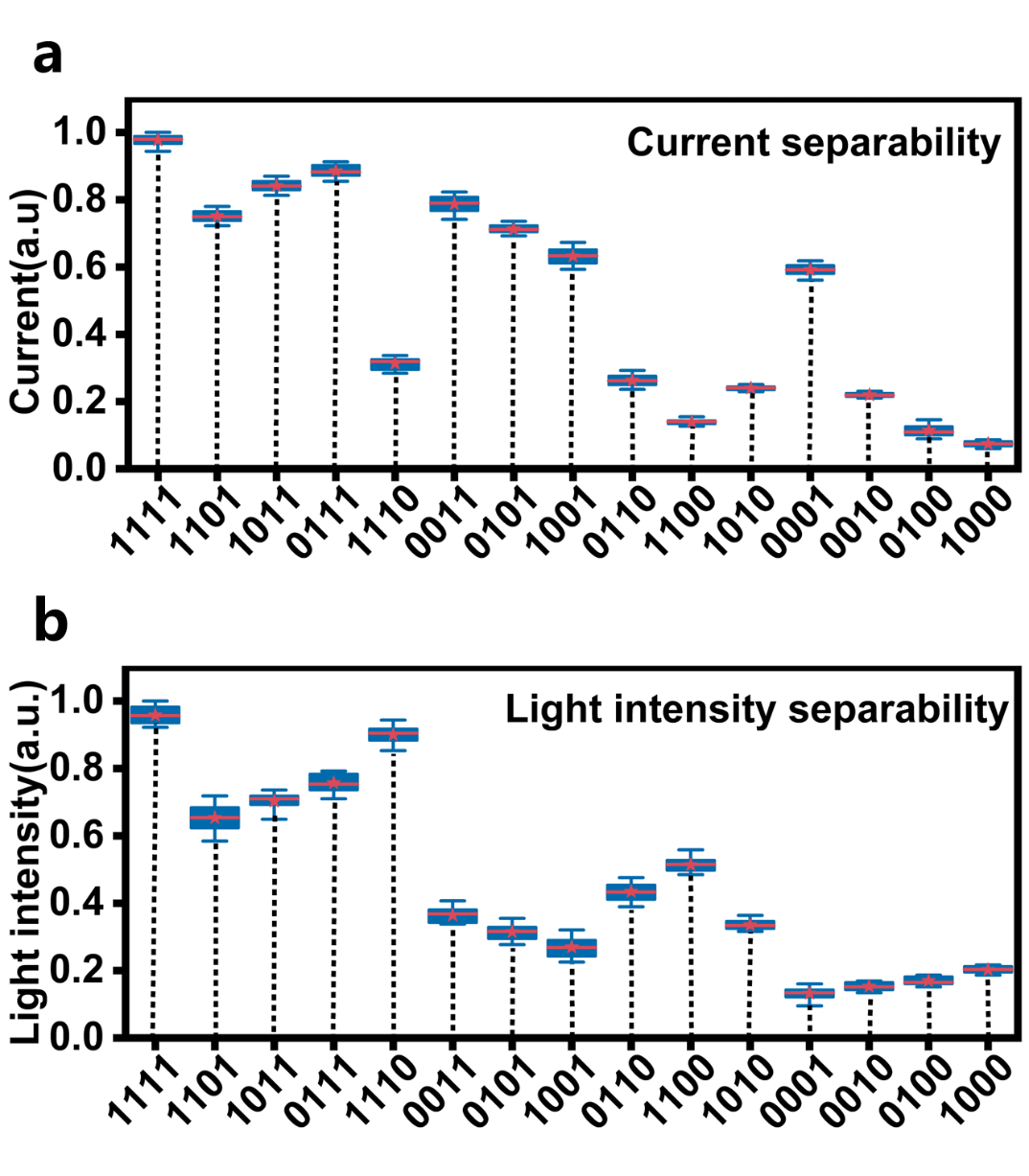


**Fig.S12. Experimental read-current responses and photo-responses**

**a** Experimental read-current responses generated by fifteen 4-bit electrical pulse trains (6 v,90 ms) ranging from (0001) to (1111). Each data point is the average of 40 repeated tests. All statistical data are presented as a mean ±SD (n=40).

**b** Experimental photo-responses generated by fifteen 4-bit electrical pulse trains ranging from (0001) to (1111). Each data point is the average of 40 repeated tests. All statistical data are presented as a mean ±SD (n=40).


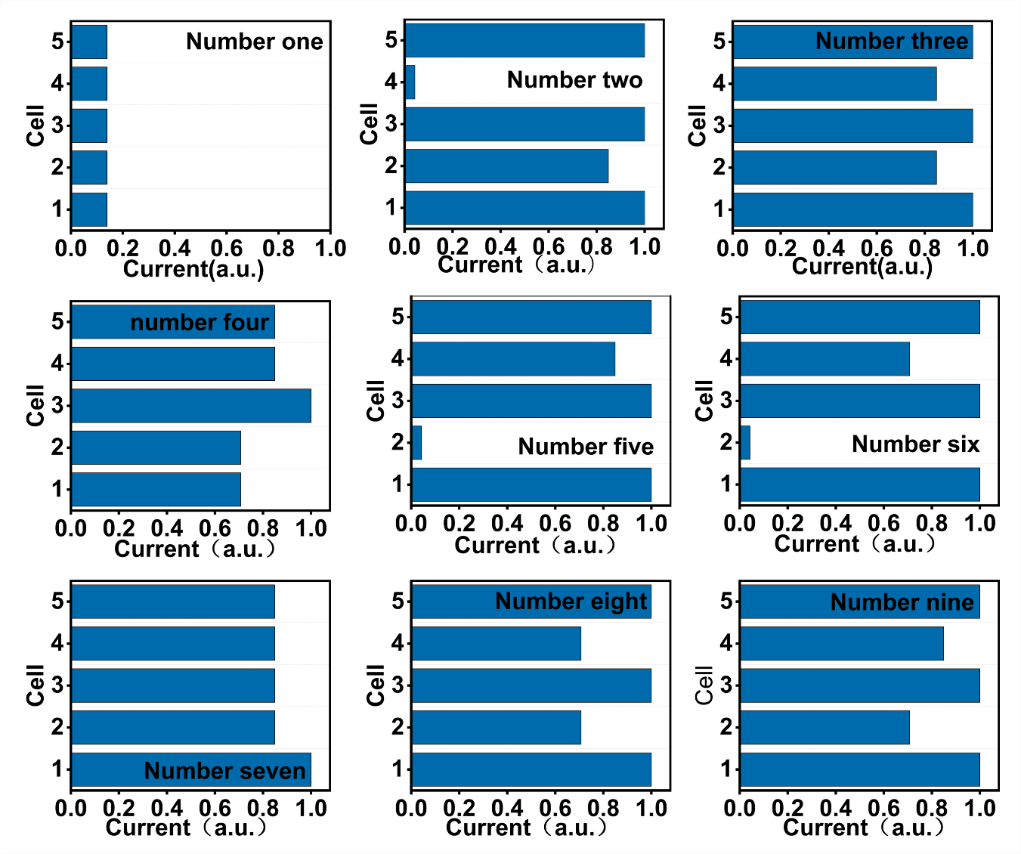


**Fig.S13. The electrical reservoir states for input number “1”to “9”.**


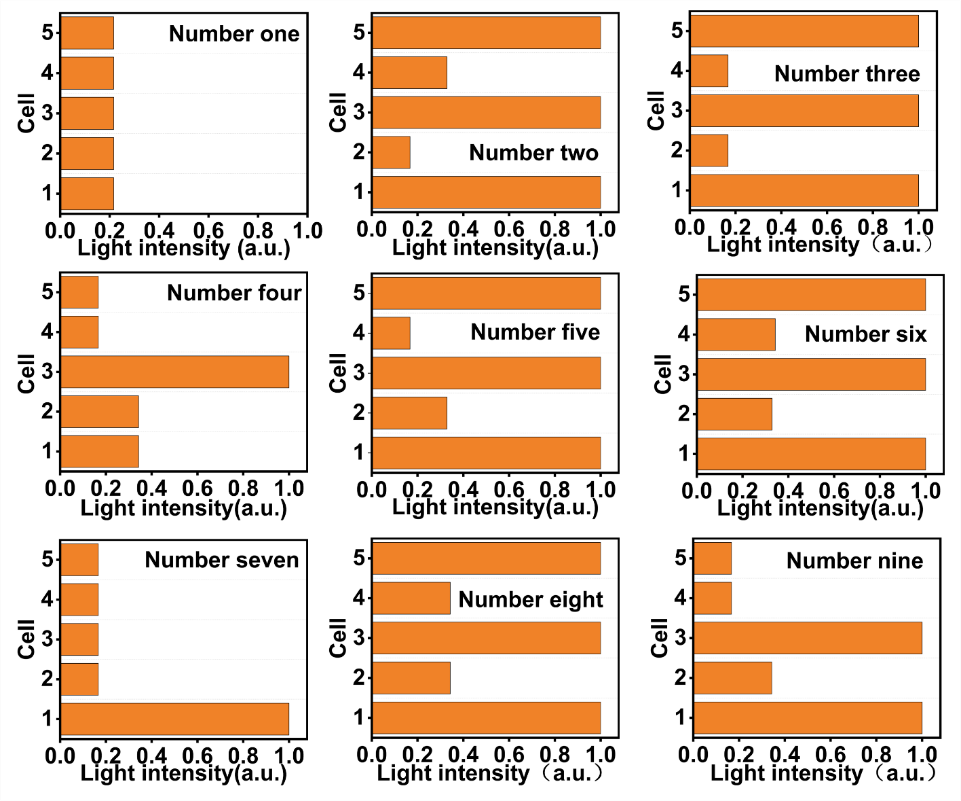


**Fig.S14. The** **light intensity reservoir states for input number “1”to “9”.**


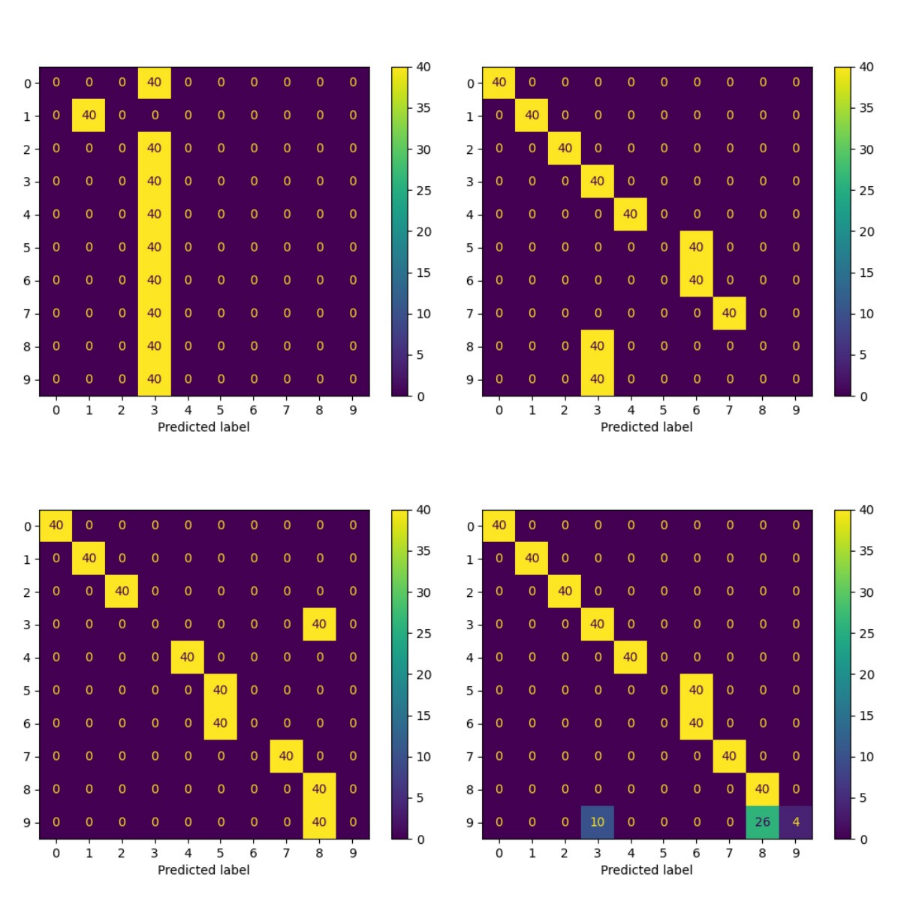


**Fig.S15. A single physical node reservoir recognizes 0-9 digital images through a confusion matrix of 1,5,10,20 cycles.**

**
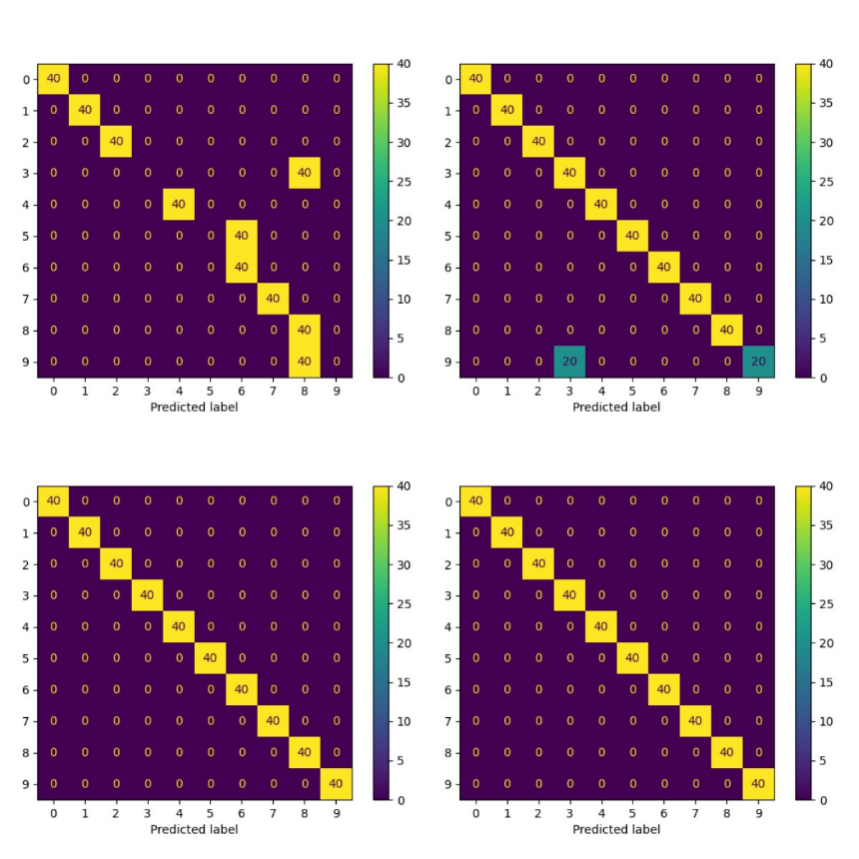
**

**Fig.S16. The mixed physical node reservoir recognizes 0-9 digital images through a confusion matrix of 1,5,10,20 cycles.**


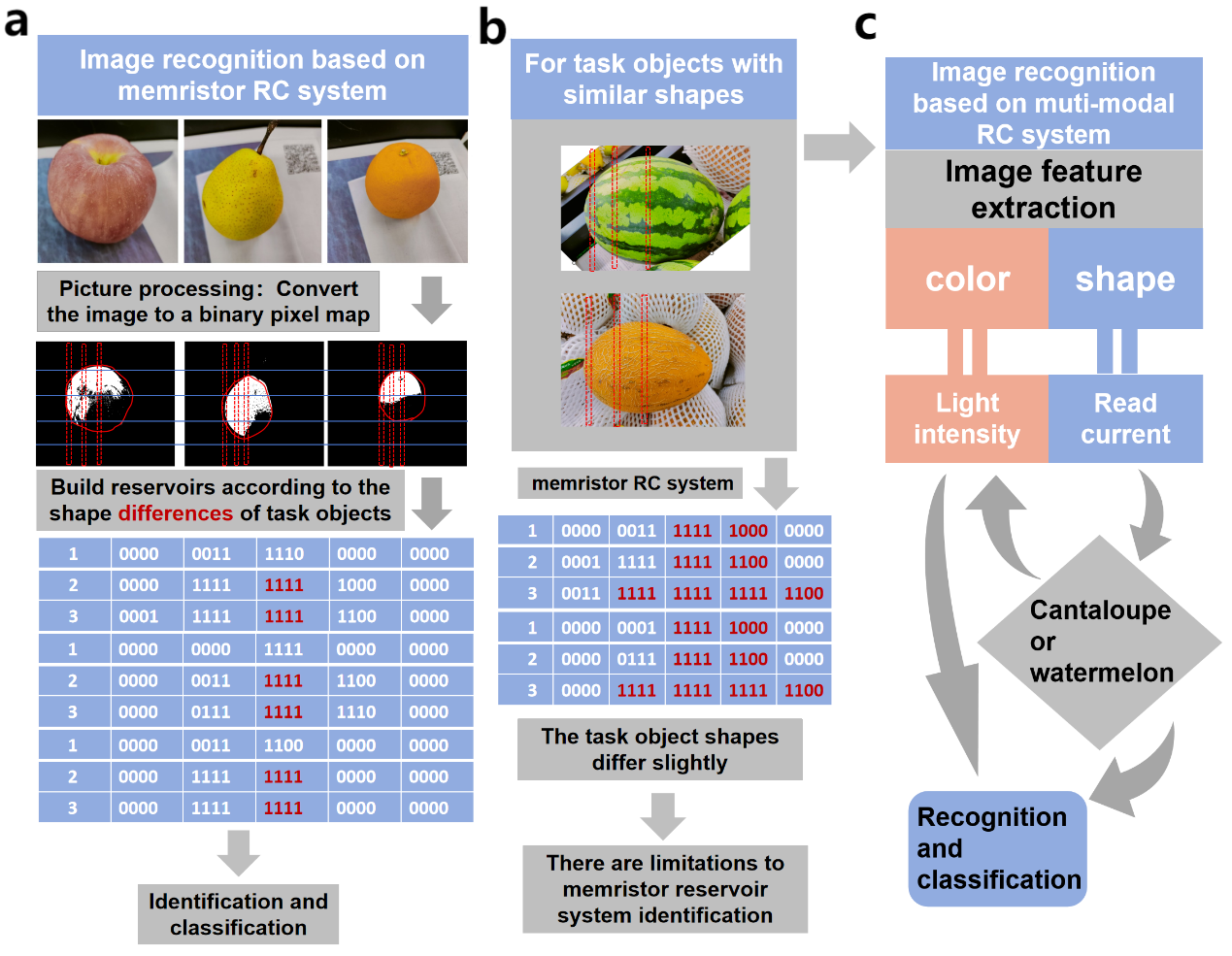


**Fig.S17. Training and recognition of the multichannel image learning.**

**a** Recognition of different images based on memristor RC system: After the images of apples, pears and oranges are preprocessed, the pixel columns are converted into pulse sequences and input into the memristor to generate distinguishable reservoirs for identification and classification.

**b** Recognition of similar images based on memristor RC system: After the watermelon and cantaloupe images are preprocessed, the pixel rows are converted into pulse sequences and input to the memristor, and the two kind of reservoir similarity is high.

**c** Schematic diagram of similar image processing based on multi-mode RC.


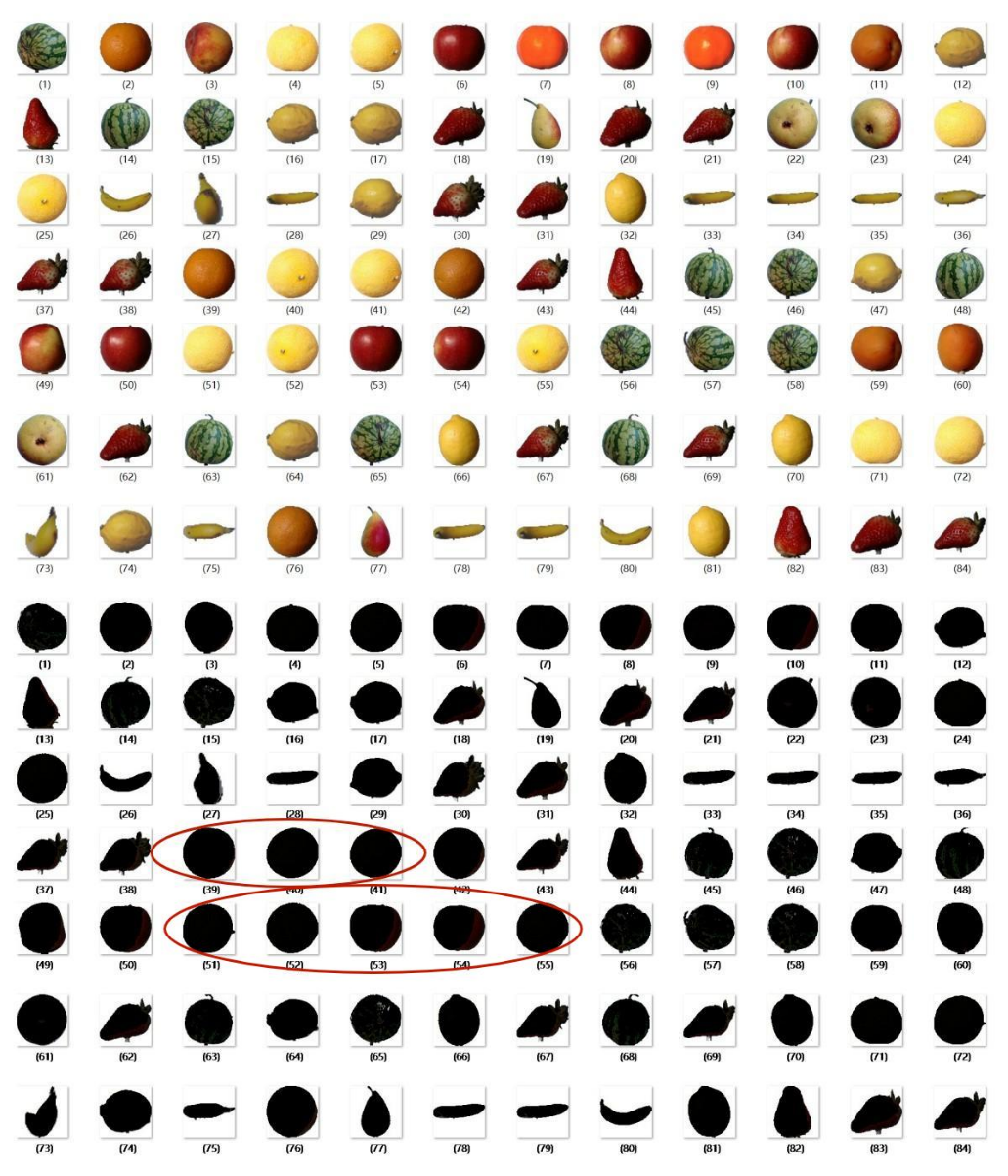


**Fig.S18. Fruit picture training set and its grayscale pictures.**


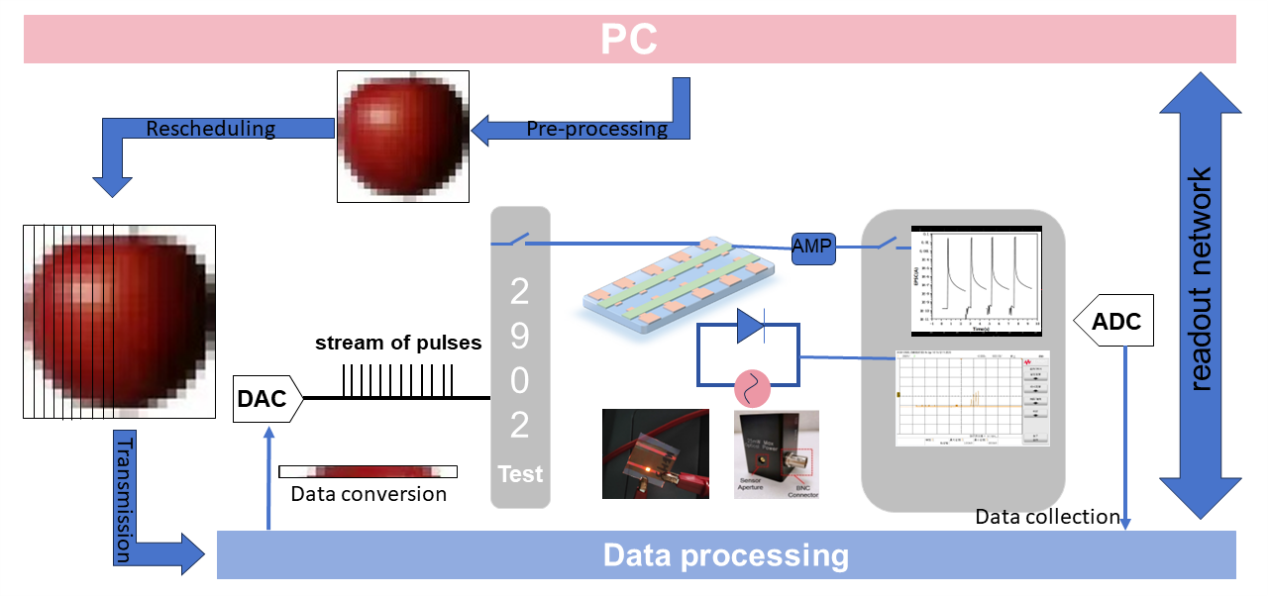


**Fig.S19. Operational flowchart of the proposed RC system for image classification.**


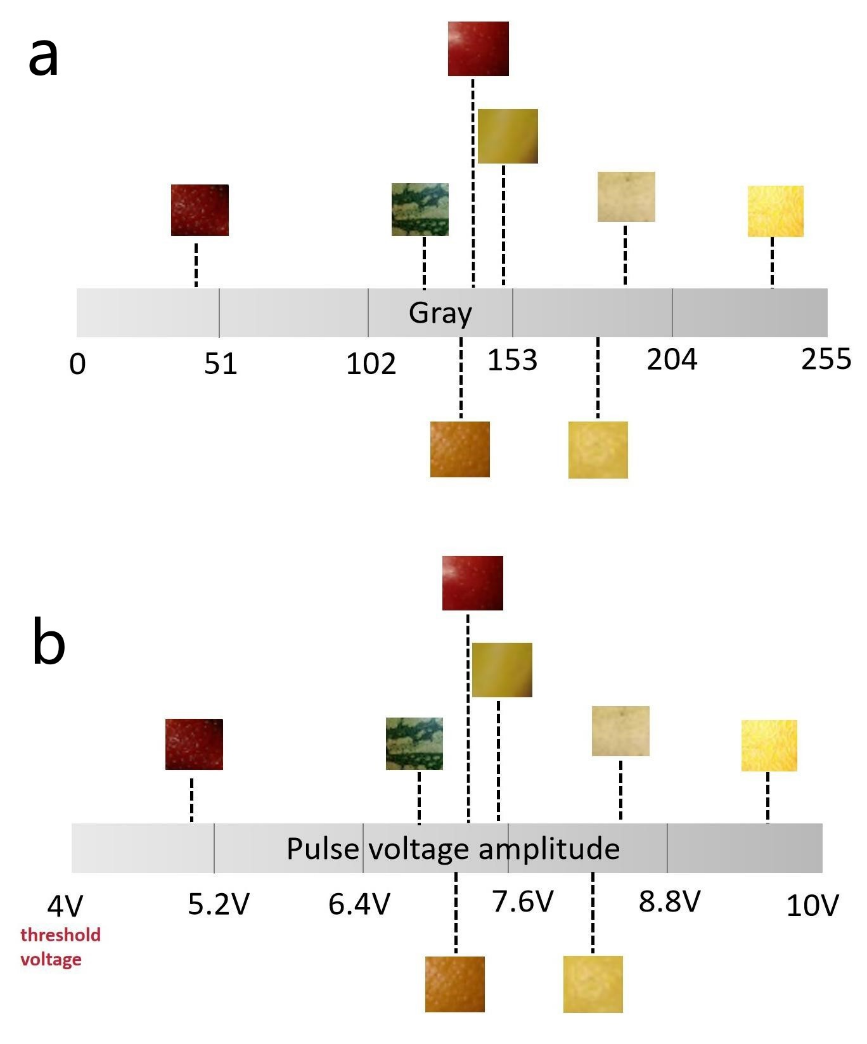


**Fig.S20. By linearly mapping grayscale values [0,255] to voltage amplitude values [4 V, 10 V].**

**a** the gray values of 8 kinds of fruits in the training set and the test set are obtained by the gray scale calculation formula:
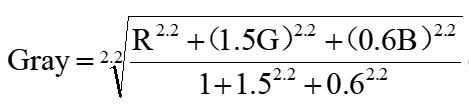
.

**b** the amplitude of the pulse voltage is set by the size of the corresponding gray value of different fruits.

**Supporting information Note 1. The detailed method for converting a handwritten digital picture into a training set.**

The handwritten digital pictures are binarized by matlab (the specific code is shown in **Fig.S21**), and the binarized data set shown in **Fig.S22** is obtained. Then, 16bit electrical reservoir state and light intensity reservoir state are obtained by experimental test (**Fig. 3d** in the manuscript), and the binarized data set is replaced by code operation (**Fig.S23**). Finally, the training set and test set for training and identification are obtained.


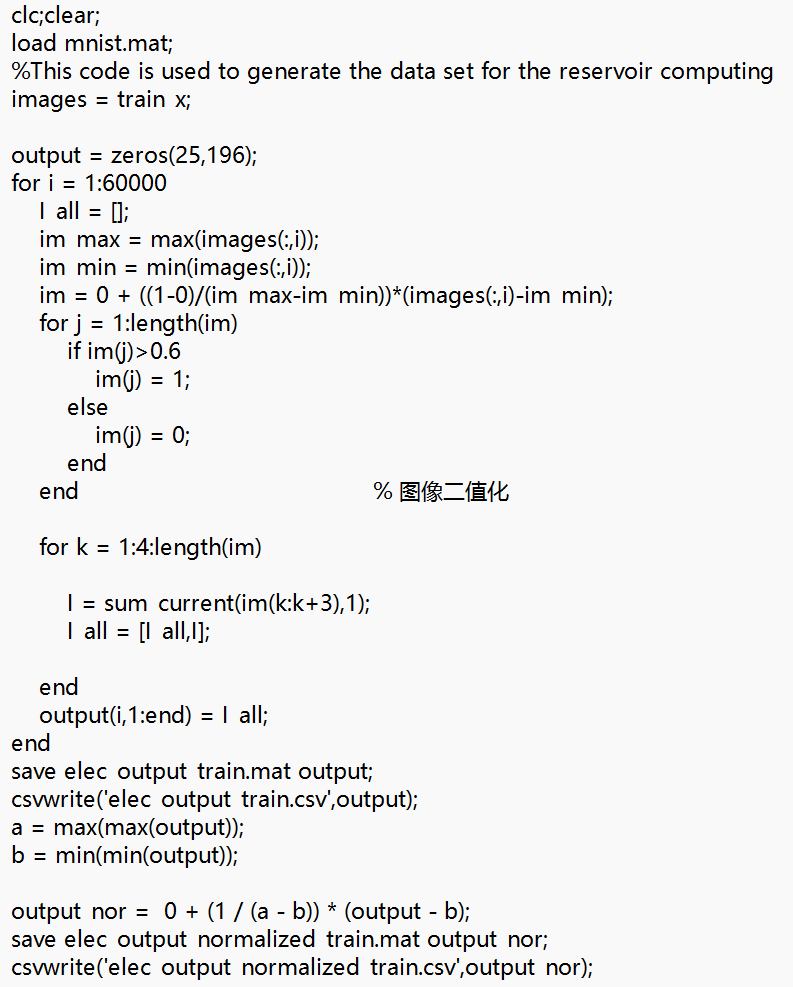


**Fig.S21. Code of handwritten digital image binarization**


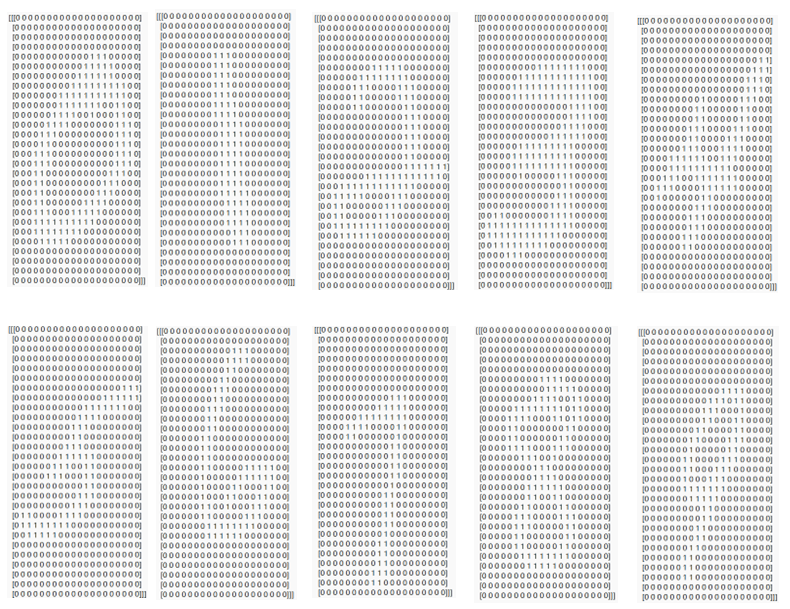


**Fig.S22. Binarization of handwritten digital images**


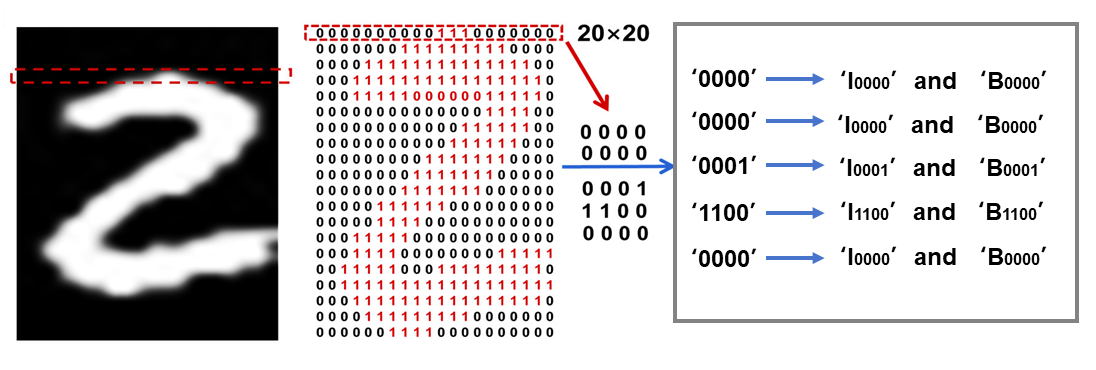


**Fig.S23. The process of replacing handwritten numeric binarization data with electrical reservoir state I and light intensity reservoir state B**

**Supporting information Note 2. The mathematical model describes the relationship between reservoir calculation and the physical device in the paper.**

As shown in **Fig.S24**, the reservoir computing network is a type of recursive neural network model based on a delayed feedback system. Its core idea is to process input signals through a fixed, sparse, randomly connected recurrent network called the reservoir. In this network, nodes receive input signals and their own feedback signals, and then update their states based on pre-set connection weights and non-linear activation functions. Afterwards, the state of the reservoir is mapped to the output layer using linear output weights, and a simple algorithm in the output layer is used to predict the temporal signal. Therefore, the relationship between the input I and the output O can be described by the following mathematical equation:

$$x(t+1)=f\left(W_{\text{res }}*x(t)+W_{\text{in }}*u(t)\right)$$

$$y(t)=W_{\text{out }}*x(t)$$

In this model, x(t) represents the current network state, x(t+1) represents the next network state. u(t) represents the current input. y(t) represents the current output. Wres is the recursive weight matrix of the reservoir, used to control the update of the current network state. Win is the input weight matrix, which used to control the influence of input signals on the state vector. Wout is the output weight matrix, used to map the state vector of the reservoir to the output. f() represents a non-linear function.Therefore, in order to enable recursion in the network, delayed feedback is required. In physical reservoir computing, physical nodes need to physically connect, transmit and respond to delayed signals, resulting in a series of virtual node behaviors.

This can be described by the following equation:

$$\frac{dx(t)}{dt}=F(t,x(t),x(t-\tau))$$

$$\theta=\tau/N$$

Where τ is the duration of the delay, N is the number of nodes, θ is the time step, and F() is a system function that depends on the physical system. Therefore, it can be observed that at the physical device level, meeting the mathematical architecture requirements of RC involves semiconductor devices having output states that exhibit non-linear response characteristics, short-term memory characteristics, and a large number of reservoir states. The number of reservoir states is highly dependent on the first two requirements.

For non-linear response, as shown in **Fig.S25,** the device exhibits non-linear response behavior. This behavior can be mathematically expressed by fitting the data as follows:

$$I=I_{(t-1)}+A\left[1-\frac{\exp\left(-\left(t-t_{0}\right)\right)}{\tau}\right]$$

$$B=B_{(t-1)}+A\left[1-\frac{\exp\left(-\left(t-t_{0}\right)\right)}{\tau}\right]$$

Where I(t-1) and B(t-1) represent the initial current state and the initial light intensity state , A is the difference between I(∞)/B(∞) and I(t-1)/ B(t-1), which is an intrinsic characteristic of the device and represents the feedback intensity, and τ is the rise time characteristic. This mathematical equation describes the evolution process of the reservoir state in the physical reservoir after being influenced by the input signals. It can be viewed as a reservoir state space. Therefore, it can be observed that there is a significant relationship between the number of reservoir state spaces and the feedback intensity A. In order to have as many reservoir state spaces as possible and to provide a wide range of reservoir states, it is necessary for the feedback intensity A to be adjustable within a large range.

On the other hand, for the short-term memory characteristic, as shown in **Fig.S26**, it can be mathematically expressed by fitting the data as follows:

$$I(t)=I_{\text{spike }}+D_{1}\exp(-t/\tau)$$

$$B(t)=B_{\text{spike }}+D_{1}\exp(-t/\tau)$$

Where, τ represent the characteristic time of the fast decayprocess, respectively. D_1_ represent the prefactor. I_spike_ and B_spike_ represent the current constant and light intensity brightness constant. Where τ is the time characteristic of the device, which is an intrinsic parameter of the device. It determines the decay rate of the memory current, thus directly affecting the state of the reservoir, the nonlinear dynamics on which the evolution of signals in the reservoir state space relies.


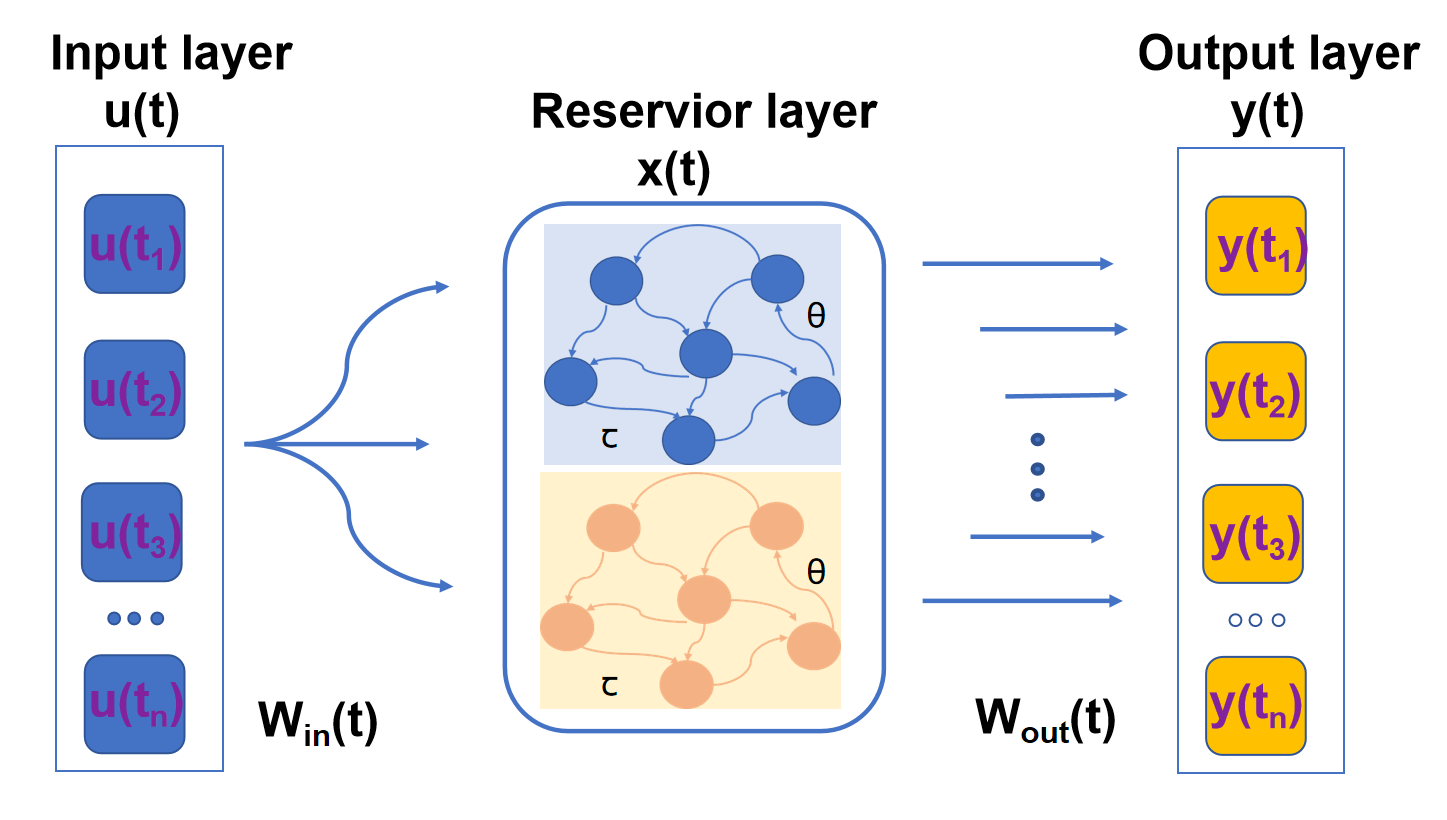


**Fig.S24. Model diagram of reservoir computing network based on delayed feedback system**


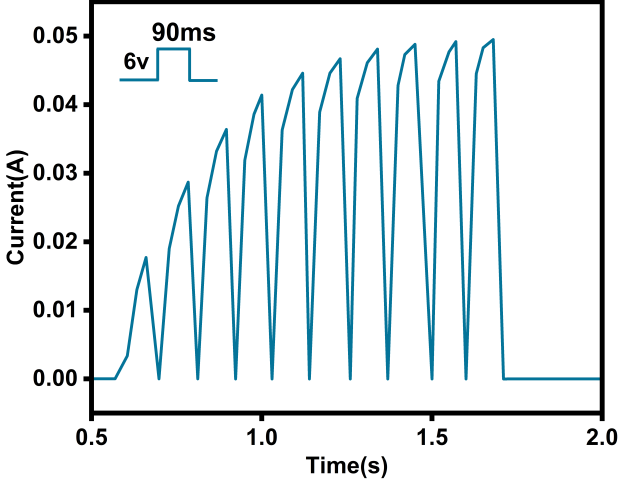

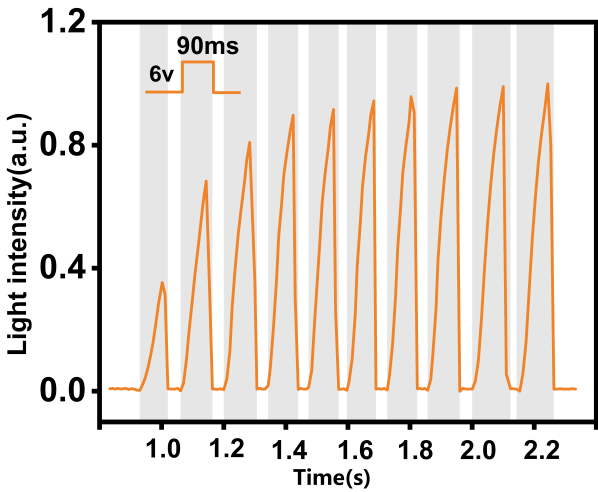


**Fig.S25. Nonlinear function extracted from the current response curve and the light response curve of the device**


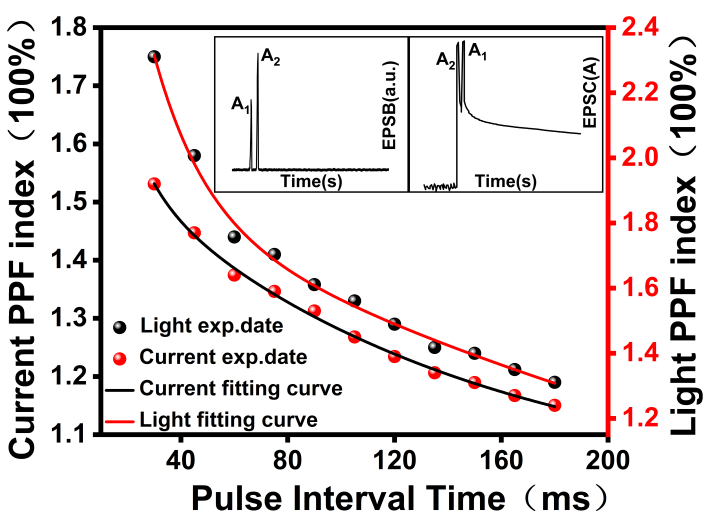


**Fig.S26. Current and light intensity PPF index (A_2_/A_1_×100%, solid sphere) as a function of the presynaptic spike internal Δt, the continuous line is the result of fitting using a single exponential decay function.**
